# Identification and validation of immune-related gene signature models for predicting prognosis and immunotherapy response in hepatocellular carcinoma

**DOI:** 10.3389/fimmu.2024.1371829

**Published:** 2024-06-12

**Authors:** Zhiqiang Liu, Lingge Yang, Chun Liu, Zicheng Wang, Wendi Xu, Jueliang Lu, Chunmeng Wang, Xundi Xu

**Affiliations:** ^1^ Department of General Surgery, The Second Xiangya Hospital of Central South University, Changsha, China; ^2^ Department of Musculoskeletal Oncology, Fudan University Shanghai Cancer Center, Shanghai, China; ^3^ Department of Oncology, Shanghai Medical College, Fudan University, Shanghai, China; ^4^ Department of General Surgery, South China Hospital of Shenzhen University, Shenzhen, China

**Keywords:** hepatocellular carcinoma, machine learning, predictive model, immunotherapy efficacy, immune checkpoint inhibitors

## Abstract

**Background:**

This study seeks to enhance the accuracy and efficiency of clinical diagnosis and therapeutic decision-making in hepatocellular carcinoma (HCC), as well as to optimize the assessment of immunotherapy response.

**Methods:**

A training set comprising 305 HCC cases was obtained from The Cancer Genome Atlas (TCGA) database. Initially, a screening process was undertaken to identify prognostically significant immune-related genes (IRGs), followed by the application of logistic regression and least absolute shrinkage and selection operator (LASSO) regression methods for gene modeling. Subsequently, the final model was constructed using support vector machines-recursive feature elimination (SVM-RFE). Following model evaluation, quantitative polymerase chain reaction (qPCR) was employed to examine the gene expression profiles in tissue samples obtained from our cohort of 54 patients with HCC and an independent cohort of 231 patients, and the prognostic relevance of the model was substantiated. Thereafter, the association of the model with the immune responses was examined, and its predictive value regarding the efficacy of immunotherapy was corroborated through studies involving three cohorts undergoing immunotherapy. Finally, the study uncovered the potential mechanism by which the model contributed to prognosticating HCC outcomes and assessing immunotherapy effectiveness.

**Results:**

SVM-RFE modeling was applied to develop an OS prognostic model based on six IRGs (CMTM7, HDAC1, HRAS, PSMD1, RAET1E, and TXLNA). The performance of the model was assessed by AUC values on the ROC curves, resulting in values of 0.83, 0.73, and 0.75 for the predictions at 1, 3, and 5 years, respectively. A marked difference in OS outcomes was noted when comparing the high-risk group (HRG) with the low-risk group (LRG), as demonstrated in both the initial training set (*P <*0.0001) and the subsequent validation cohort (*P <*0.0001). Additionally, the SVMRS in the HRG demonstrated a notable positive correlation with key immune checkpoint genes (CTLA-4, PD-1, and PD-L1). The results obtained from the examination of three cohorts undergoing immunotherapy affirmed the potential capability of this model in predicting immunotherapy effectiveness.

**Conclusions:**

The HCC predictive model developed in this study, comprising six genes, demonstrates a robust capability to predict the OS of patients with HCC and immunotherapy effectiveness in tumor management.

## Introduction

Hepatocellular carcinoma (HCC), a predominant subtype of primary liver cancer, constitutes approximately 75–85% of all such cases and ranks as one of the most prevalent malignancies affecting the digestive system on a global scale (1). Data from CLOBOCAN 2020 reveal that the global annual incidence of new liver cancer cases per year has increased to 905,677 (1). Correspondingly, it is ranked the sixth most common form of malignancy. With a death toll of 830,1.8 million, it is also ranked third in terms of mortality (1). In addition, a persistent increase is projected in the number of new liver cancer cases, potentially establishing it as the foremost cause of cancer-related deaths in many countries (2). However, patients with early-stage HCC can improve their chances of survival by undergoing radical treatment. Nonetheless, it is important to note that even with this aggressive approach, the 5-year recurrence rate remains as high as 70% (3). Hence, facilitating the identification of patients at risk of unfavorable clinical outcomes is essential for the prompt detection of recurrence and metastasis in HCC. Such progress is key for timely treatment and mitigation of the disease burden on patients with HCC.

At present, immune checkpoint inhibitors (ICIs) are widely used to treat patients with advanced HCC. In this context, two extensive clinical trials have demonstrated that combining ICIs and VEGFR-targeted drugs is superior to sorafenib in treating patients with advanced HCC (4, 5). Among these studies, the IMbrave150 trial highlighted that administering a combination therapy of atezolizumab (PD-L1 inhibitor) and bevacizumab outperformed sorafenib in augmenting overall survival (OS) and progression-free survival (PFS) outcomes in individuals with advanced, unresectable HCC when utilized as the first-line treatment (4). The ORIENT-32 trial, which assessed the effectiveness of combining Sintilimab (PD-1 inhibitor) with IBI305 (bevacizumab biosimilar) in treating unresectable HBV-associated HCC in a Chinese patient cohort, revealed notable improvements in both OS and PFS compared to the sole administration of sorafenib as a first-line treatment (5). Thus, the pivotal challenge lies in pinpointing biomarkers capable of accurately predicting the response of patients with HCC to ICIs. Such identification is essential to protect patients from unbeneficial therapies and to diminish both healthcare and financial burdens (6).

Our study initially screened immune-related genes (IRGs) associated with patient prognosis, followed by the application of various machine learning techniques for modeling. Once the optimal model was selected, its clinical application value was validated using our cohort and multiple data sets from various sources. Thus, the primary objective was to establish a model that can be utilized for prognostic evaluation to precisely diagnose HCC, while also predicting adverse clinical outcomes and offering timely intervention. Furthermore, the model demonstrated a notable potential in predicting the efficacy of immunotherapy, with an objective to categorize the immunotherapeutic responses in patients with HCC and to aid in the optimization of clinical pharmacotherapy.

## Materials and methods

### Data acquisition and processing

The TCGAbiolinks package (version: 2.28.4) (7) in R (version: 4.2.2) software was utilized to retrieve liver hepatocellular carcinoma (LIHC) patient data, including clinical information and gene expression spectrum matrix, from The Cancer Genome Atlas Program (TCGA) database (https://portal.gdc.cancer.gov/) on November 15, 2022. The training set consisted of 305 samples with HCC, PFS, and OS data for a period of at least 30 days. These samples had complete clinical stage information, prognostic follow-up information, and expression profile matrix. The transcript abundance data measured in transcripts per kilobase (TPM) and the gene symbol table associated with the ENSID were acquired separately. When there were multiple matches between the gene symbol and ENSID, the median value of expression data was selected. Additionally, genes with no expression (TPM = 0) in more than half of patients with LIHC were excluded.

In addition, the DNA methylation- and RNA-based stemness score (RNAss), was obtained from the UCSC Xena database (https://xenabrowser.net/datapages) (8) on December 11, 2022. This data was used for the subsequent analysis of tumor stemness correlation in the training sets.

The validation datasets of this study consisted of 54 patients who were diagnosed and treated for stage I-IV HCC between January 1, 2015, and December 31, 2016. These patients were identified based on strict admission criteria using pathology. The primary inclusion criteria were as follows: 1) initial diagnosis made at our hospital; 2) absence of any other malignancies; 3) availability of complete follow-up data. OS was delineated as the duration from the initial identification of HCC in a patient to the point of either their death or the conclusion of the follow-up period. The follow-up concluded on December 31, 2021, incorporating outpatient consultations and telephone-based follow-ups. The present research received approval from the Clinical Research Ethics Committee of the Second Xiangya Hospital, Central South University (Approval No. LYF2022070). Prior to hospitalization, all patients had provided their explicit consent by signing informed consent forms. Another independent ICGC−LIRI−JP HCC cohort data were extracted from 231 patients with HCC and their corresponding clinical information in the International Cancer Genome Consortium (ICGC) database (https://dcc.icgc.org/).

Additional three validation datasets containing gene expression and clinical information were obtained from TIGER (http://tiger.canceromics.org) (9) on March 20, 2023. These datasets, which are independent of the main dataset, include three cohorts: Melanoma-phs000452 (10), non-small cell lung cancer (NSCLC)-GSE135222 (11), and renal cell carcinoma (RCC)-Braun_2020 (12). Specifically, the Melanoma-phs000452 cohort involved 153 patients receiving an anti-PD-1 drug, the NSCLC-GSE135222 cohort had 27 patients undergoing treatment with an anti-PD-1 drug, and the RCC-Braun_2020 cohort consisted of 311 patients who received treatment with a combination of anti-PD-1 and Everolimus drugs. The IRGs were singled out from the ImmPort database (accessible at https://www.immport.org/shared/genelists) (13) on December 20, 2022. A total of 1793 IRG were identified after eliminating duplicate genes.

### Univariate survival analysis

To pinpoint the genes implicated in the OS and DFS among patients in the training set, a univariate survival analysis was carried out using the Survival package (version 3.3–1) in the R 4.2.2 software environment. Next, the two gene sets mentioned above were compared with IRG. After identifying the overlapping genes, the expression matrix of these genes was extracted from the verification set for further modeling. The survival curve was generated utilizing the ggsurvplot function from the survminer package (version: 0.4.9). The optimal threshold for gene classification was established using the surv_cutpoint function, which facilitated classifying genes into high- and low-expression groups based on this threshold. Subsequently, the hazard ratio (HR), along with its 95% confidence interval (CI) and P-values for the genes incorporated in the model, were graphically depicted using the forestploter package (version 1.0.0).

### Logistic and least absolute shrinkage and selection operator regression analysis

In medical research, logistic regression stands out as a crucial statistical method for analyzing the intricate relationship between diseases and their pathogenic factors, providing valuable insights into the underlying mechanisms of disease development (14). LASSO regression, on the other hand, offers the advantage of flexibility in handling both continuous and discrete dependent variables with minimal data requirements, making it widely applicable, while also facilitating variable selection and reducing model complexity (15).

Following the processing of the gene expression profile and patient survival data, Logistic and LASSO regression models were built using the glmnet package (version: 4.1–6) (16, 17). Among them, logistic regression was employed to model the survival status of patients, using it as the dependent variable. The regression analysis involved the use of glm and step functions, with the direction set as “both”. Finally, variables with a significance level of *P <*0.05 were selected for the construction of the logistic regression model. In the LASSO regression analysis, the family was set to “binomial”, alpha was set to “1”, nfolds was set to “10”, and lambda.1se was selected based on the coef function the acquisition of the regression coefficients of each gene. Subsequently, the LASSO regression model was then constructed based on this selection. The risk scores of the two aforementioned models were obtained using the predict function and designated as the logistic regression risk score (LRRS) and the LASSO-associated risk score (LARS), respectively.

### Support vector machine-recursive feature elimination analysis and modeling

SVM-RFE, as an embedded method widely utilized in pattern recognition and machine learning, demonstrates its practical value by effectively employing structural risk minimization to enhance learning performance, utilizing a sequential backward selection algorithm to iteratively refine feature sets, and ultimately enabling the construction of prognostic models and analysis of immunotumor microenvironment correlations through targeted gene screening (18, 19). The significant variables identified in the logistic regression analysis were combined with the variables used in constructing the LASSO regression model. The resulting Venn diagram was generated using the Vennerable package (version 3.0). After integrating the above-mentioned variables with the patient survival information, the caret package (version: 6.0–94) (20) was used for the Recursive Feature Elimination (RFE); herein, the function was set to “caretFuncs”, the method was set to “cv”, and the number was set to “10”. After filtering out the optimal factors for modeling, SVM modeling was conducted using the e1071 package (version 1.7–14), the type was specified as “C-classification”, and the kernel was set to “radial”. The decision values of this model were utilized as risk scores and designated as support vector machine risk score (SVMRS).

### Model evaluation

Cutoff values for risk scores across the three models were established through the surv_cutpoint function in the survminer package. Based on these values, patients were categorized into high-risk group (HRG) and low-risk group (LRG). The risk scores and groupings from the three models were then combined with the patient data for further evaluation of the models.

To assess the differentiation of the aforementioned models, we utilize the cindex function from the pec package (version: 2023.04.12) (21) for both evaluation and visualization purposes. Subsequently, decision curve analysis (DCA) was conducted utilizing the rmda package (version 1.6) to ascertain the clinical net benefit derived from the three models. The predictive performance of each mode was examined by computing the area under the curve (AUC) values at three different time intervals: 1 year, 3 years, and 5 years. For these calculations, the timeROC package (version: 0.4) (22) was utilized, and the results were visually represented through the receiver operating characteristic (ROC) curves. In addition, the confusion matrices were examined and graphed using the yardstick package (version 1.2.0). To assess the model’s ability to accurately recall patients who experienced a fatal clinical outcome, we utilized modEvA (version: 3.9.3) (23) to generate precision-recall curves (PRC) and calculate their AUC values. Finally, to evaluate the level of calibration of the models, the calibration curves and nomogram diagrams were drawn using the calibrate functions and nomogram functions of the rms package (version: 6.5.0). The evaluation and comparison of all the aforementioned differentiations and calibrations were consolidated to establish the ultimate prognostic model suitable for the study cohort. The risk factor correlation diagrams, ROC curves, and survival curves were generated based on the risk score, patient survival, survival time, and gene expression of each model.

### Correlation analysis between SVMRS or IRGs and clinical pathological features of TCGA-LIHC

From the clinical information of TCGA-LIHC patients, a representative set of features, including stage, comorbidities, and Eastern cooperative oncology group (ECOG) performance scores, were selected. We then analyzed whether these clinical pathological features exhibited significant differences and correlations between patient groups based on SVMRS values and the TPM expression levels of the six IRGs utilized in the construction of models.

### Real-time quantitative polymerase chain reaction and validation of the prognostic value of models

For RT-qPCR analysis, total RNA from 54 liver tissue samples (from patients in our cohort) embedded in paraffin was isolated utilizing the BIOG RNA FFPE Tissue Kit in accordance with the guidelines specified by Baidai (Changzhou, China). The synthesis of cDNA was accomplished utilizing the Evo M-MLV RT Mix kit complemented with gDNA Clean (Accurate Biotechnology, Hunan, China). To ascertain the SVMRS, the detection of the expression of the genes to be tested was conducted through qPCR utilizing the SYBR^®^ Green Pro Taq HS qPCR KIT (Accurate Biotechnology, Hunan, China). The gene expression levels were standardized using the 18S rRNA as a reference. The primers and their corresponding sequences are documented in **Supplementary Table S1**. We utilized the SVMRS (Support Vector Machine Regression Score) of each patient, along with their prognosis and survival information, as well as gene relative expressions in our cohort. Subsequently, the risk factor correlation diagrams, ROC curves, and survival curves were generated to validate the prognostic significance of the model. The aforementioned analytical approaches were also utilized in the independent ICGC−LIRI−JP HCC cohort to validate the prognostic predictive value of the model.

### Tumor stemness and immune cell infiltration analysis

The data from the TCGA database exhibited a positive association between the stemness score of HCC and unfavorable clinical outcomes in patients. This finding implies a notable correlation of the tumor stemness score with the OS and PFS in the context of TCGA-LIHC (24). Consequently, we examined the disparities in six tumor stemness scores between the HRG and LRG. This comparison was done as per the tumor stemness scores derived from 305 samples and the corresponding risk groups of patients in the training set. Moreover, a prominent association was identified between the stemness scores and the tumor immune microenvironment (TIME) (24). Consequently, the distribution of 22 different types of immune cells in the training set was analyzed using the CIBERSORT package (version: 0.1.0) (25). Subsequently, we examined the variations in immune cell types between groups based on the HRG and LRG of patients.

### Analysis and validation of immunotherapy efficacy prediction

The genes examined in this study were IRG, which may possess specific prognostic significance for immunotherapy effectiveness. To substantiate this hypothesis, we initially examined the variations in expression levels of four frequently utilized immunotherapy drug targets: CTLA-4, PDCD1 (PD-1), CD274 (PD-L1), and PDCD1LG2 (PD-L2), between HRG and LRG. Subsequently, the expression correlations between SVMRS and the aforementioned four genes were examined based on the classification into HRG and LRG. This analysis aimed to make an initial assessment of the potential immune prediction value.

Subsequently, we employed three distinct validation cohorts that had undergone immunotherapy. These cohorts were then classified into HRG and LRG using SVM-RFE modeling following the same methodology. The study then focused on examining the differences in SVMRS between the HRG and LRG, with an emphasis on evaluating the immunotherapy responses in the validation cohorts. The predictive performance of the model for the immunotherapy responses was further verified by conducting survival analyses in conjunction with the prognostic information of patients.

### Functional enrichment and pathway analysis

To delve into the mechanistic underpinnings of differentiating the HRG from the LRG, we initially analyzed the differentially expressed genes (DEG) using the limma package (version: 3.56.2) (26). This investigation was carried out with a fold change threshold of “2” and a false discovery rate (FDR) of “0.05”. The list of DEGs was used to conduct Gene Ontology (GO) enrichment analysis through the application of the clusterProfiler package (version: 4.8.3) (27, 28). The data were graphically depicted using the GOplot package (version 1.0.2) (29). Subsequently, the gene list and fold change value were utilized to conduct gene-set enrichment analysis based on the Kyoto Encyclopedia of Genes and Genomes (GSEA-KEGG). A threshold of *P <*0.05 was set to ascertain the statistical significance of the results. Visualization of the GSEA results was achieved through the dotplotGsea function in the GseaVis package (version: 0.0.9) and the gseaNb function from the same package. Additionally, the cnetplot function from the enrichplot package (version: 1.20.3) was used for visualization.

The identified pathways of interest were retrieved from the PathCards database (https://pathcards.genecards.org/) (30). The expression matrix of these genes was extracted and used for expression correlation analysis with SVMRS. Each gene was analyzed individually. The ComplexHeatmap software (version 2.16.0) (31, 32) was used for visualization.

### Statistical analysis

The data in this study underwent statistical analysis using GraphPad Prism software (version 9.0.0, San Diego, California, USA) for both statistical analysis and image rendering. The software package of the method utilized default parameters for the parameters that were not specified. Additionally, the ggplot2 package (version: 3.3.5) (33) was employed for data visualization, which was not explicitly mentioned. Spearman correlation analysis was used for correlation analysis. The scatterplots were generated using Sangerbox (http://www.sangerbox.com/tool) (34). Additionally, the study utilized the Mann-Whitney rank sum test for the analysis of continuous variables of skewed distribution between two groups. In contrast, when data conformed to a normal distribution with consistent variance, the Student’s t-test was utilized for executing a comparative analysis between the two groups. For the comparative analysis of multiple sets of data that satisfy the assumptions of homogeneity of variance and normal distribution, Ordinary one-way ANOVA should be employed, followed by Holm-Šídák’s multiple comparisons test for pairwise comparisons within the groups. However, if these assumptions are not met, the Kruskal-Wallis test should be utilized for the comparison among multiple groups, accompanied by Dunn’s multiple comparisons test for within-group comparisons. For discrete variables, the Chi-square test was used for comparison between groups. A significance level of *P <*0.05 was used to determine statistical significance (*P <0.05, **P <0.01, ***P <0.001, ****P <0.0001).

## Results

### The SVM-RFE model was developed using 6 prognosis related genes

**Figure 1:**
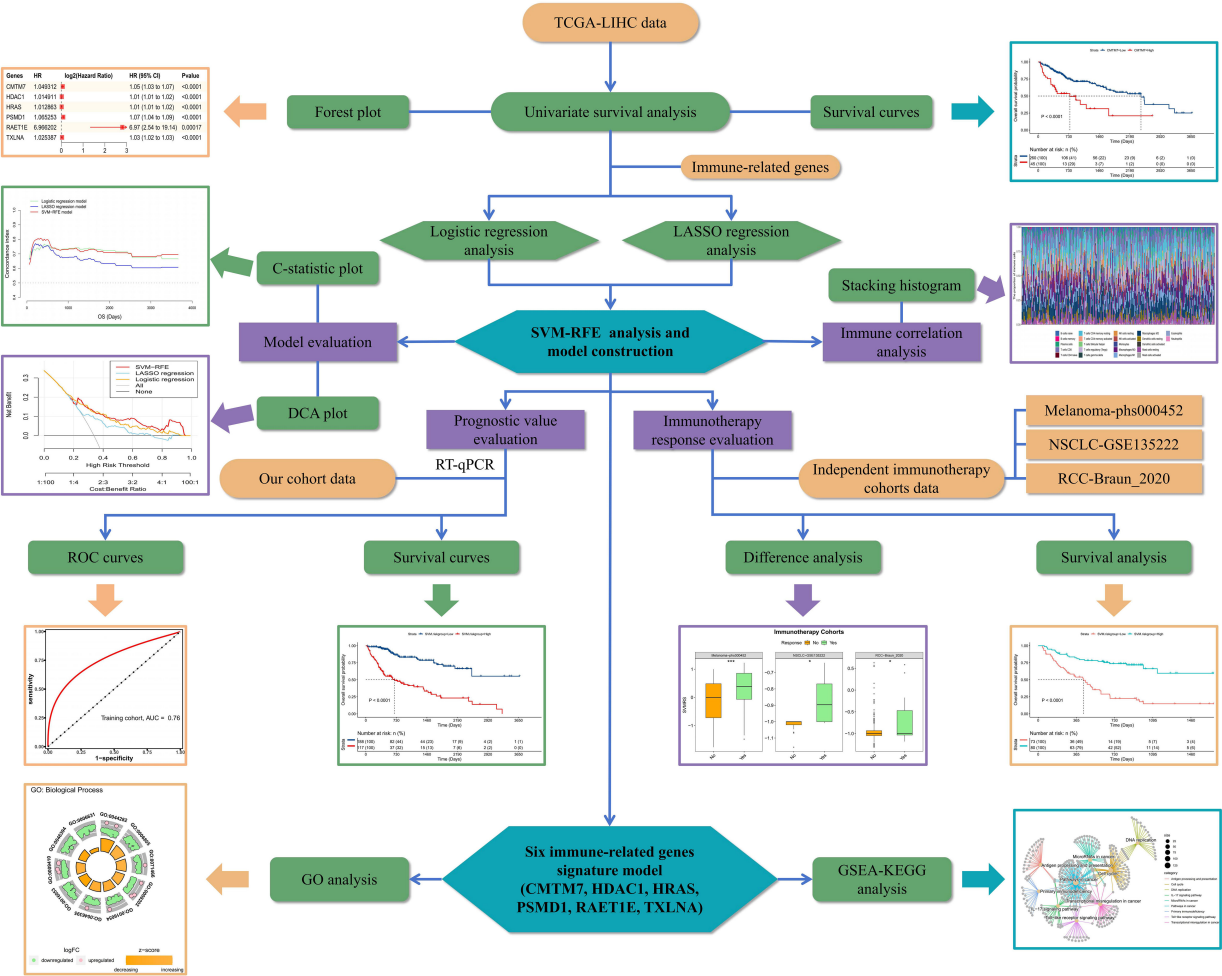
Flow chart of this study.

**Figure 2:**
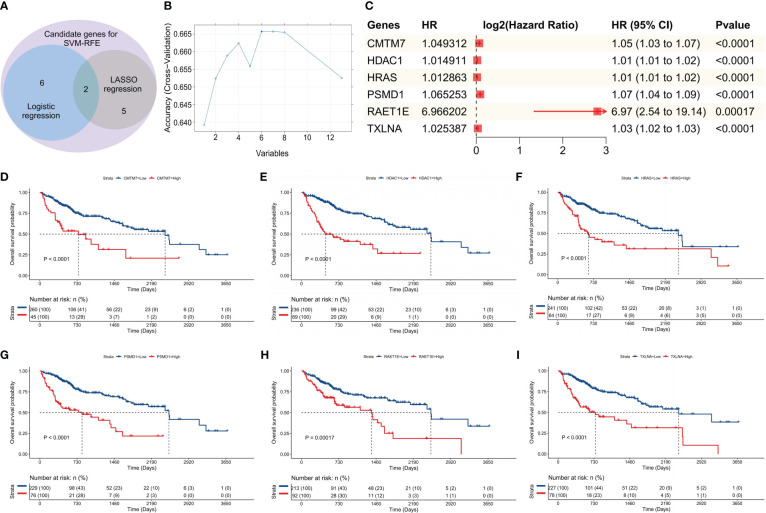
Construction of the SVM-RFE model. **(A)** Venn diagram of the genes included in the three models during the initial screening; **(B)** Line chart for the change in accuracy with the increase in variables during the analysis; **(C)** Univariate survival analysis forest plot, based on the gene symbol, HR, 95% CI, and P value; D–I. Survival curves plotted as per the optimal cut-off value for each gene group; the genes were: **(D)** CMTM7, **(E)** HDAC1, **(F)** HRAS, **(G)** PSMD1, **(H)** RAET1E, and **(I)** TXLNA, respectively.

The analytical flow chart for this study is shown in **Figure 1**. As observed, the univariate survival analysis revealed that there were 6608 genes influencing PFS and 9772 genes influencing OS in the training set. Furthermore, 81 genes were obtained after the intersection with IRG, which were used for subsequent modeling (**Supplementary Figure S1**). Additionally, logistic regression analysis identified eight genes, while LASSO regression analysis screened seven genes. Among these, two genes were found to be common to both methods. Therefore, a total of 13 genes were selected for SVM-RFE modeling (**Figure 2A**). Following the RFE analysis, a total of six genes were selected to be included in the construction of the final model (**Figure 2B**). The HR, 95% CI, and P-values for these genes in the univariate analysis are shown in **Figure 2C**, demonstrating that all six genes were identified as risk factors. Moreover, these six genes were identified as prognostic markers and were found to have an impact on the OS of patients in TCGA-LIHC. The specific genes are as follows: CMTM7 (*P <*0.0001, HR = 1.05, **Figure 2D**), HDAC1 (*P <*0.0001, HR = 1.01, **Figure 2E**), HRAS (*P <*0.0001, HR = 1.01, **Figure 2F**), PSMD1 (*P <*0.0001, HR = 1.07, **Figure 2G**), PAET1E (P = 0.00017, HR = 6.97, **Figure 2H**), TXLNA (*P <*0.0001, HR = 1.03, **Figure 2I**).

### SVM-RFE model was found to be the best model in this study

**Figure 3:**
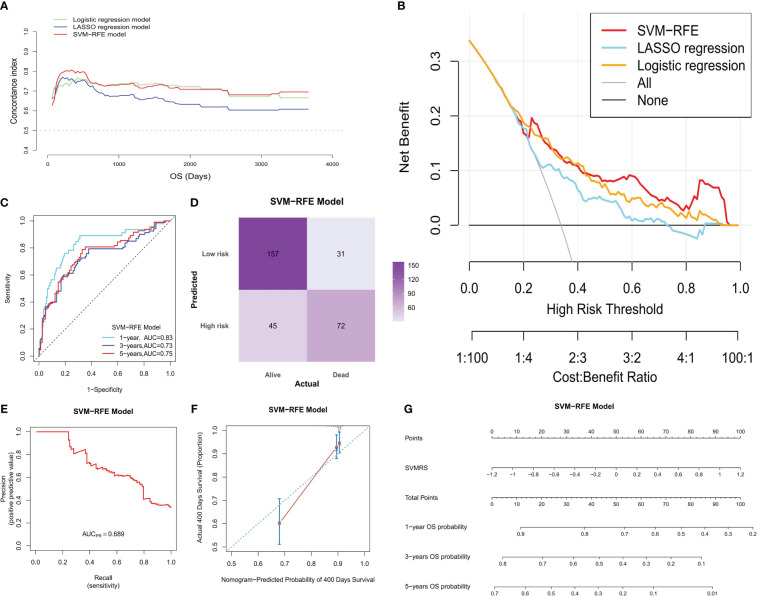
Model evaluation. In the training set: **(A)** The C-statistic (vertical coordinate) was plotted with the patient’s OS (horizontal coordinate) changes; **(B)** DCA of the three models; the horizontal coordinate represents the risk threshold, and the vertical coordinate represents the net benefit. **(C)** The ROC curves were drawn based on the risk score of the SVM-RFE model and the 1-, 3- and 5-year OS time recorded. **(D)** The confusion matrix was plotted according to the classification of HRG and LRG of patients by the model, combined with the actual death status of patients; **(E)** The PRC was drawn according to the accuracy and recall rate of the model; **(F)** The calibration curve of the SVM-RFE model was plotted at 400 days (the time point comprised the best calibration degree). The horizontal coordinate denotes the predicted survival situation, and the vertical coordinate denotes the actual survival situation. Every 100 people were divided into groups and resampled 1000 times. **(G)** Nomogram developed according to the risk score, the total points, and its corresponding 1-, 3-, and 5-year OS probability.

As per the aforementioned outcomes, the SVM-RFE model exhibits the advantage of having a limited number of constituent genes. Thus, to further verify the optimal nature of this model in our study, we conducted additional evaluations focusing on differentiation and clinical applicability. Upon analyzing the fluctuation of the C-statistic in relation to the OS time, we determined that the SVM-RFE model exhibited the highest level of effectiveness (**Figure 3A**). Similarly, the DCA outcomes indicated that all three models were capable of enhancing the net benefit, with the SVM-RFE model exhibiting the greatest increase in net benefit (**Figure 3B**). Additionally, the time-dependent ROC curve analysis revealed that the SVM-RFE model achieved AUC values of 0.83, 0.73, and 0.75 for the 1-, 3-, and 5-year OS predictions, respectively (**Figure 3C**). In addition, the SVM-RFE model was found to outperform both the logistic regression model (**Supplementary Figure S2A**) and the LASSO regression model (**Supplementary Figure S2B**). Furthermore, the accuracy of the SVM-RFE model (75.08%) was higher (**Figure 3D**) than that of the logistic regression model (70.16%, **Supplementary Figure S2C**), as well as the LASSO regression model (69.51%, **Supplementary Figure S2D**). Meanwhile, we constructed the Precision-Recall Curve (PRC) to evaluate the efficacy of these three models in accurately identifying dead samples. As observed, the use of the logistic, LASSO, and SVM-RFE models improved the probability of detecting dead cells from an initial 33.77% (103/105) to 63.2% (**Supplementary Figure S2E**), 52.3% (**Supplementary Figure S2F**), and 68.9% (**Figure 3E**), respectively, with the SVM-RFE model having the highest precision-recall rate among the three models. Thus, the SVM-RFE model proved to be the most effective model in this study. In addition, the risk score of the model also indicated a high goodness of fit (**Figures 3F, G**).

### The model has potential value in prognostic prediction

**Figure 4:**
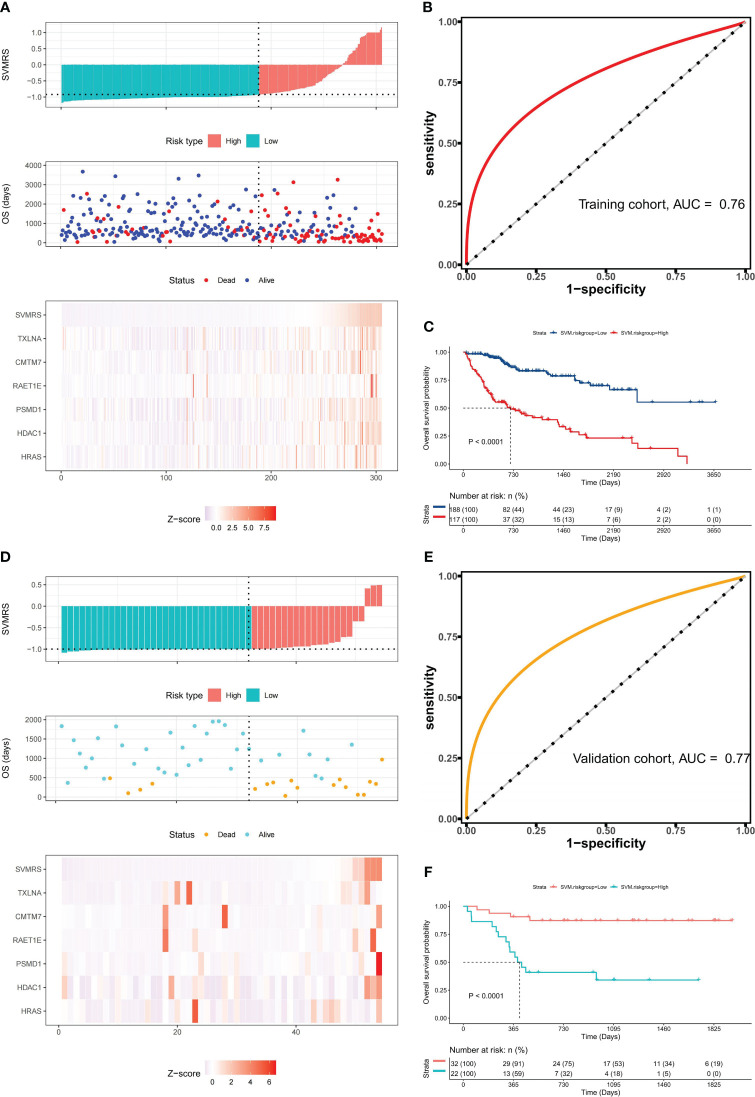
Prognostic value of the model. **(A)** Risk factor association diagram of the model in the training set includes a histogram of the high and low distribution of the patient’s risk score, the scatter plot of the patient’s survival situation distribution, and the heat map of the change of gene expression value with the associated risk scores. The horizontal coordinate represents the number of patients ranked by risk score from low to high; the ordinates represent risk score, OS, and model-related genes. **(B)** ROC curve drawn as per the risk score calculated by the model and the survival state of the patient in the training set; **(C)** Survival curve was drawn as per the optimal value of the risk score (SVMRS = -0.9214) in the training set. **(D)** Risk factor correlation diagram of the model in our cohort; **(E)** ROC curve based on the model’s risk score and the survival status of patients in our cohort. **(F)** Survival curve drawn by the HRG and LRG as per the optimal value of the risk score (SVMRS = -0.9981) in our cohort.

By calculating the Survival-associated Variable Model Risk Score (SVMRS) for each patient and integrating the survival status and gene expression values, risk factor association diagrams were generated to assess the prognostic prediction of the risk score for the 305 patients. **Figure 4A** demonstrates the arrangement of patients based on their risk scores, ranging from low to high. The optimal cut-off value for SVMRS (-0.9214) was employed to classify patients into HRG and LRG. The mortality rate in the HRG was significantly higher than that in the LRG, and all the genes with elevated expression levels were exclusively found in the HRG, indicating that these genes were all associated with increased risk. Additionally, the ROC curve demonstrated an AUC value of 0.76 for this model’s ability to predict patient mortality in the training set (**Figure 4B**). Furthermore, a statistically noteworthy contrast in the OS rate was found between the HRG and LRG (**Figure 4C**). This result indicates that individuals with an elevated SVMRS risk score were more prone to unfavorable outcomes.

Similarly, the prognostic performance of this model was verified using our validation cohort. As indicated by the risk factor diagram (**Figure 4D**), all six genes were confirmed to be risk factors. In addition, the ROC curve of our validation cohort indicated that the model accurately predicted patient mortality, as evidenced by an AUC value of 0.77 (**Figure 4E**). The results of survival analysis also demonstrated a significantly poorer clinical prognosis in the HRG compared to the LRG (**Figure 4F**). In the independent ICGC−LIRI−JP HCC cohort, we also observed consistent results with those mentioned previously. Specifically, the risk factor diagram (**Supplementary Figure S3A**), ROC curves (**Supplementary Figures S3B, C**), survival curve (**Supplementary Figure S3D**), and confusion matrix (**Supplementary Figure S3E**) all indicated that the model effectively stratified patients into risk groups and accurately predicted their OS. Thus, based on the analysis and validation conducted, it can be concluded that this model holds promise in predicting OS in patients with HCC.

### SVMRS and the six IRGs were correlated with selected clinicopathologic features of HCC patients

Further clinical correlation analysis of SVMRS and the expression profiles of six IRGs utilized in model construction revealed that HRAS (**Supplementary Figure S4A**), PSMD1 (**Supplementary Figure S4B**), and SVMRS (**Supplementary Figure S4C**) were associated with T stage, with their values generally increasing alongside T stage progression. Additionally, PSMD1 (**Supplementary Figure S4D**) was found to be related to N stage, exhibiting significantly higher expression in the N1 group. HRAS (**Supplementary Figure S4E**), PMSD1 (**Supplementary Figure S4F**), TXLNA (**Supplementary Figure S4G**), and SVMRS (**Supplementary Figure S4H**) were associated with stage, where higher values corresponded to later stages. These findings further validate the prognostic predictive value of our constructed model.

Moreover, from the perspective of patients’ comorbidities, RAET1E (**Supplementary Figure S5A**) and SVMRS (**Supplementary Figure S5B**) were associated with comorbidities. Specifically, TXLNA was linked to hepatitis B (**Supplementary Figure S5C**), SVMRS to hepatitis C (**Supplementary Figure S5D**), and RAET1E to non-alcoholic fatty liver disease (**Supplementary Figure S5E**). Regarding ECOG performance scores, the expression levels of HDAC1 (**Supplementary Figure S5F**), PSMD1 (**Supplementary Figure S5G**), and CMTM7 (**Supplementary Figure S5H**) correlated with them, where higher scores corresponded to increased gene expression. Similarly, SVMRS demonstrated a correlation with ECOG scores, as shown in **Supplementary Figure S5I**. This suggested that higher SVMRS values are associated with comorbidities and increased ECOG scores, indicating a poorer prognosis for patients with HCC. The clinical correlation analysis of risk grouping exhibited high consistency with SVMRS, except for hepatitis C, where no significant statistical differences were observed. However, the differences in T stage, overall Stage, comorbidities, and ECOG scores aligned with SVMRS (**Supplementary Table S2**), suggesting the feasibility of the risk grouping approach employed in this study.

### Differences in RNAss and immune cell infiltration between HRG and LRG

**Figure 5:**
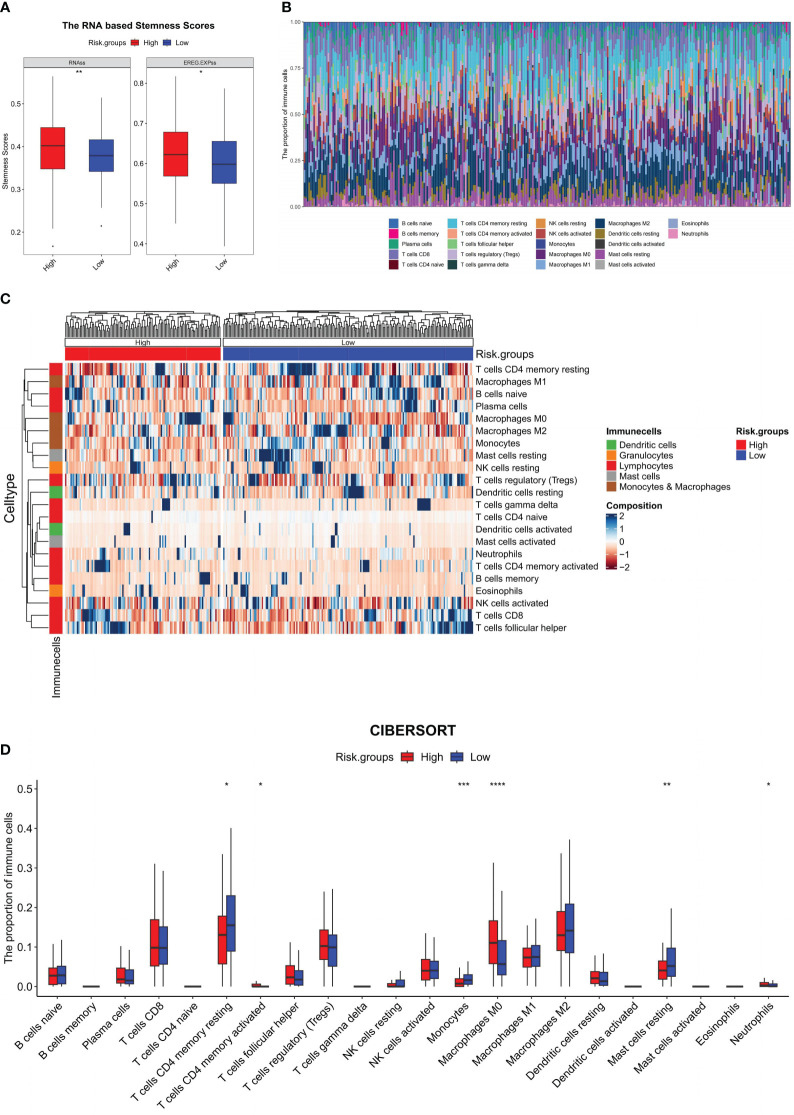
Immune microenvironment analysis of the model. In the training set: **(A)** RNA-based stemness scores were analyzed between HRG and LRG and represented using a boxplot; **(B)** Stacking histogram representing the proportion of 22 types of immune cells; **(C)** Heat map representing the proportion of 22 kinds of immune cells between the HRG and LRG; **(D)** Boxplot for the difference of 22 types of immune cells between the HRG and LRG; *P <0.05, **P <0.01, ***P <0.001, ****P <0.0001.

The investigation into tumor stemness unveiled a noteworthy difference in the RNAss and the epigenetically regulated RNAss (EREG.EXPss) between the HRG and LRG. Accordingly, the values of RNAss (P = 0.0035) and EREG.EXPss (P = 0.0217) were prominently augmented in the HRG compared to the LRG (**Figure 5A**). Given the correlation between this value and the TIME, we proceeded to perform a detailed analysis of the differences in the proportion of 22 different types of immune cell subtypes between the two groups. The distribution of immune cells in 305 samples in the training set was depicted in **Figure 5B**, while the comparison of proportions between the HRG and LRG was illustrated in **Figure 5C**. The analysis comparing HRG and LRG revealed that the LRG exhibited a higher proportion of T cell CD4 memory resting (P = 0.0113), monocytes (P = 0.0003), and mast cells resting (P = 0.0055). Moreover, the HRG exhibited a higher proportion of T cells CD4 memory activated (P = 0.0415), macrophages M0 (*P <*0.0001), and neutrophils (P = 0.0264) (**Figure 5D**).

### The model has potential value in predicting immunotherapy response

**Figure 6:**
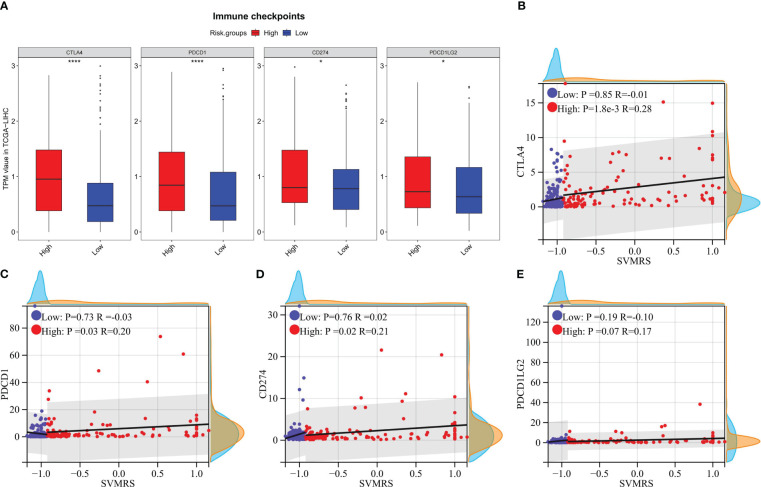
Correlation analysis between risk score and the expression of ICGs. In the training set: **(A)** The differences of 4 commonly used ICGs between HRG and LRG were analyzed and shown using a boxplot; B–E. Scatter plots for the correlation analysis between SVMRS and **(B)** CTLA-4, **(C)** PD-1, **(D)** PD-L1, and **(E)** PD-L2 in the HRG and LRG. *P <0.05, ****P <0.0001.

The outcomes of the analytical investigations revealed significant differences in immune checkpoint gene (ICG) expression between HRG and LRG. Specifically, the expression levels of four widely utilized immunotherapy drug targets, *viz.*, CTLA-4 (*P <*0.0001), PD-1 (*P <*0.0001), PD-L1 (P = 0.0124), and PD-L2 (P = 0.0182), were notably higher in the HRG compared to the LRG (**Figure 6A**). In addition, the correlation analysis indicates that CTLA-4 (P = 0.0018, R = 0.28, **Figure 6B**), PD-1 (P = 0.03, R = 0.20, **Figure 6C**), and PD-L1 (P = 0.03, R = 0.21, **Figure 6D**) exhibited a significant positive correlation with SVMRS in the HRG. However, no correlation was observed in the LRG. Thus, while the statistical significance of the correlation between PD-L2 and SVMRS in HRG was not established, a noticeable trend could be observed (P = 0.07, R = 0.17, **Figure 6E**). Hence, we hypothesized that the HRG may exhibit greater susceptibility to immunotherapy.

**Figure 7:**
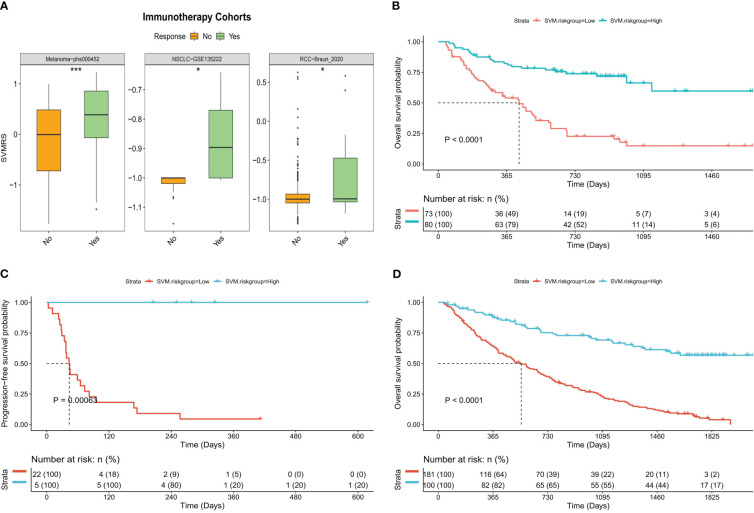
Predictive value of the model for immunotherapy response. **(A)** Boxplot of the difference of SVMRS between the response and no response group using the three immunotherapy validation datasets. **(B–D)**. Survival curve as per the patient’s risk groups and the survival status of patients in three immunotherapy validation cohorts, **(B)** Melanoma-phs000452, **(C)** NSCLC-GSE135222, and **(D)** RCC-Braun_2020; *P <0.05, ***P <0.001.

To verify the aforementioned hypothesis, three independent cohorts from different platforms were utilized as immunotherapy validation datasets for further analysis. All patients in the three cohorts received treatment with anti-PD-1 medications, and both the effectiveness of the drugs and the prognosis of the patients were recorded. The analysis of differences revealed that the SVMRS in the group of individuals who responded was significantly greater than that in the group of individuals who did not respond in the Melanoma-phs000452 cohort (P = 0.0004). Similar results were observed in the NSCLC-GSE135222 cohort (P = 0.0112) and the RCC-Braun_2020 cohort (P = 0.0236) (**Figure 7A**). Furthermore, the survival analysis demonstrated statistically significant disparities between the HRG and LRG in all three cohorts: Melanoma phs000452 (*P <*0.0001, **Figure 7B**), NSCLC-GSE135222 (P = 0.0001, **Figure 7C**), and RCC-Braun_2020 (*P <*0.0001, **Figure 7D**). Previous analyses have revealed that the HRG, which had a worse prognosis, experienced significantly longer survival after undergoing immunotherapy. This survival advantage was notably superior to that of the LRG, indicating that the HRG could derive substantial benefits from immunotherapy. Thus, it was verified that the model possesses the capability to predict immunotherapy effectiveness.

### Signaling pathways related to tumorigenesis and immune progression were activated in the HRG

**Figure 8:**
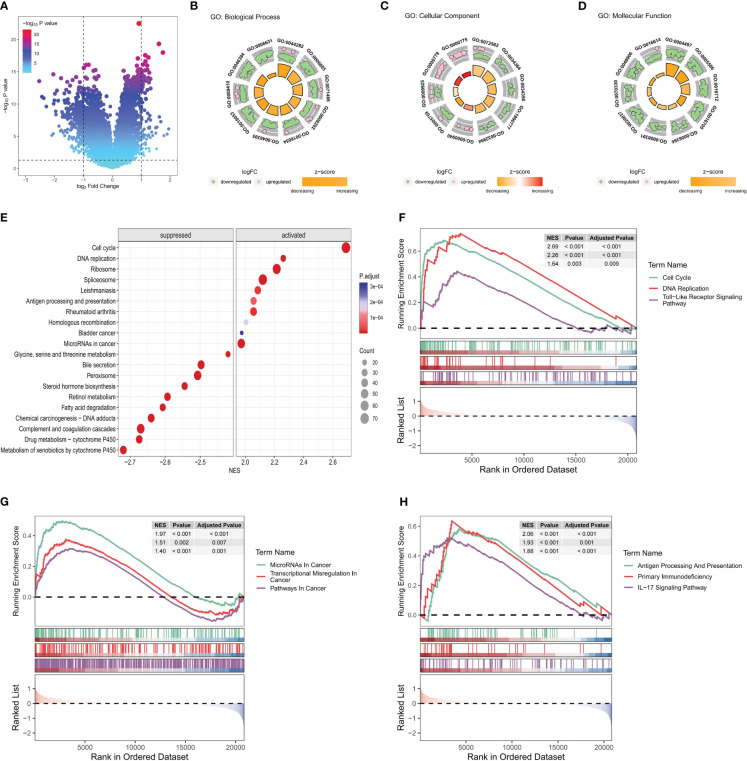
Functional enrichment analysis of key differential genes in the model. In the training set: **(A)** The volcano plot is based on risk groups for differential analysis, where the horizontal ordinate denotes the log2 Fold Change and the longitudinal coordinates denote the -log10 P value. Use |log2fold change|=1 to draw the vertical dotted line and P = 0.05 to draw the horizontal dotted line. **(B–D)** Ranked by P value, chord diagrams of the top 10 results of **(B)** biological process, **(C)** cellular component, **(D)** molecular function plotted from GO enrichment analysis of DEG. **(E)** Ordered by NES, the top 10 suppressed or activated pathways were shown according to the GSEA-KEGG pathway analysis results. **(F–H)**. Pathways associated with **(F)** tumorigenesis, **(G)** tumor progression, and **(H)** immune progression that were activated in the HRG, as selected from the GSEA-KEGG pathway analysis results.

The differential expression analysis of DEGs between HRG and LRG identified a total of 341 genes. Among these DEGs, 89 exhibited upregulated expression and 252 exhibited downregulated expression in the HRG (as shown in **Figure 8A**; **Supplementary Table S3**). Further, the GO enrichment analysis of these 341 DEGs pointed to their potential roles in the regulation of the top 10 biological processes, cell components, and molecular functions (**Figures 8B–D**). The details, as well as the corresponding GO enrichment results, are shown in **Supplementary Table S4**. As observed, these genes are found to be primarily associated with tumor metabolism. Further analysis of the GSEA-KEGG pathway revealed the top 10 pathways that were either suppressed or activated. These pathways are presented in **Figure 8E** and are ranked based on the normalized enrichment score (NES). Additionally, it is evident that the inhibited pathways exhibited an increase in metabolic activity, whereas the stimulated pathways were associated with the development of tumors and immune processes.

**Figure 9:**
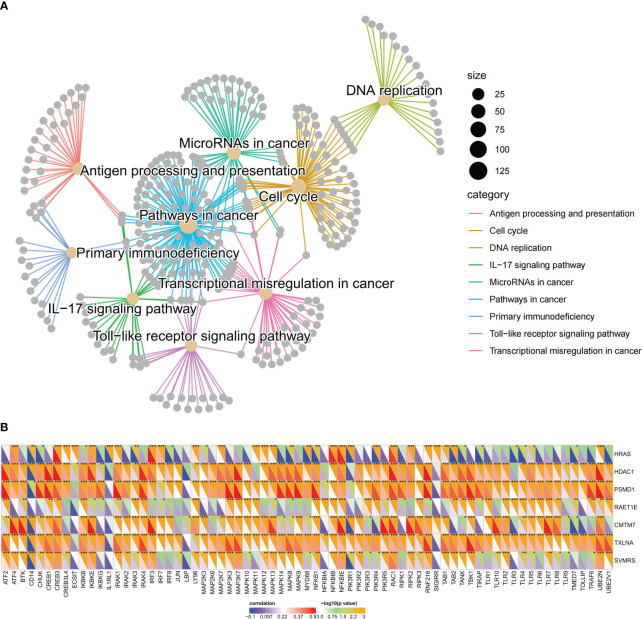
Pathways regulated by the key genes of the model. **(A)** The interaction network constructed by the nine representative pathways; each gray dot represents a gene; different pathways were represented using different colors; **(B)** SVMRS and the six genes used in modeling correlated well with the expression of key genes involved in the TLR signaling pathway. The lower left half triangle in each column represents the correlation coefficient. Blue represents a negative correlation and red represents a positive correlation; the darker the color, the stronger the correlation. The upper right half triangle represents the results of Spearman correlation analysis; *P <0.05, **P <0.01, ***P <0.001, ****P <0.0001.

Nine pathways that were activated in the HRG were selected from all the relevant pathways (as shown in **Supplementary Table S5**). The pathways associated with tumorigenesis comprised the cell cycle, DNA replication, and the Toll-like receptor (TLR) signaling pathway (**Figure 8F**). In addition, the tumor progression pathways identified were microRNAs in cancer, transcriptional dysregulation in cancer, and pathways in cancer (**Figure 8G**). Moreover, the immune-related pathways identified were antigen processing and presentation, primary immunodeficiency, and IL-17 signaling pathway (**Figure 8H**). Subsequently, visualization of the interconnection network among the aforementioned 9 exemplary pathways was performed, and a strong correlation between all 9 pathways was observed (**Figure 9A**). Finally, the study focused on examining the expression profiles of key genes involved in the TLR signaling pathway, which are associated with tumorigenesis and immune processes. Notably, a correlation analysis was conducted to examine the relationship between the SVMRS and the expression of the six genes constituting the model, as depicted in **Figure 9B**. All the key genes in the TLR signaling pathway exhibited statistically significant correlations with SVMRS or the six genes utilized in the modeling. This indicates that these key genes in the model are likely to have an immunoregulatory and cancer-promoting function by participating in the regulation of this pathway. This could also be one of the intrinsic mechanisms contributing to the unfavorable prognosis and heightened vulnerability to immunotherapy in the HRG of patients.

## Discussion

In recent years, numerous studies have focused on using sequencing data to identify markers that can impact the prognosis of patients with HCC and develop corresponding models, aiming to enhance the accuracy of patient prognosis prediction and provide guidance for clinical practice (35–37). For instance, Wang et al. (35) developed four gene signature models associated with disulfidptosis to predict OS outcomes in the context of HCC. Herein, the calculated AUC values of ROC for OS at 1, 3, and 5 years in the training set were 0.766, 0.736, and 0.699, respectively, demonstrating noteworthy potential in predicting the effectiveness of anti-tumor therapy. Furthermore, Chen et al. (36) constructed a model to predict the OS of patients with HCC using five genes related to cuproptosis. In the training set, the calculated AUC values of ROC for the model were recorded at 0.775, 0.685, and 0.670 for 1, 3, and 5 years, respectively. In addition, Shi et al. (37) have successfully developed a ten epithelial-mesenchymal transition (EMT)-related genes signature prognostic model for HCC, validating its accuracy in stratifying patients into high and low-risk groups using datasets from TCGA and International Cancer Genome Consortium (ICGC), and its risk score tightly correlates with tumor stage, grade, and immune cell infiltration, exhibiting significant prognostic value with ROC AUC values of 0.767, 0.694, and 0.680 at 1-, 2-, and 3- year OS in the training set, respectively. In our study, we developed a prognostic model based on machine learning consisting of six IRGs for predicting the survival of patients with HCC. Accordingly, the calculated AUC values of ROC for 1-, 3-, and 5-year OS were 0.83, 0.73, and 0.75, respectively, demonstrating the significance of predicting OS in patients with HCC.

All of the six key genes used to build the model were IRG, of which CMTM7, belonging to the Chemokine-like factor (CKLF)-like MARVEL transmembrane domain-containing proteins (CMTM) family, plays a crucial function in the immune system and is abundantly expressed in immune tissues (38). CMTM7 functions as a tumor suppressor in various types of cancer within the field of cancer research. For example, knockdown of CMTM7 was observed to impair the process of autophagy and accelerate the development of tumors in lung cancer (39). Moreover, CMTM7 was also found to serve as a potential biomarker for identifying immunological traits and predicting immunotherapy effectiveness in breast cancer (40). On the other hand, HDAC1 is critically involved in regulating gene expression by modulating the acetylation of both histone and non-histone proteins (41). Correspondingly, its overexpression has been frequently associated with the progression, metastatic potential, and prognostic outcomes of multiple cancer types, including colon, gastric, prostate, and breast cancers. In addition, HDAC1 is linked to unfavorable prognosis and resistance to chemotherapy in cases of pancreatic cancer (42). Moreover, HDAC inhibitors exhibit potent anticancer effects in hematological malignancies and hold promise as potential therapeutic agents for treating colorectal cancer (43) and triple-negative breast cancer (44). HRAS also comprises a prevalent oncogene, which is positioned upstream of the RAS/MAPK signaling pathway and plays a pivotal role in transmitting signals from the extracellular environment to the nucleus, leading to cell growth, division, proliferation, and differentiation (45). HRAS mutations have the ability to trigger YAP1-AXL signaling, leading to metastasis in head and neck cancer (46). Moreover, HRAS overexpression in gastroenteropancreatic neuroendocrine tumors is strongly associated with a notable response to lenvatinib (47). Additionally, PSMD1 is classified as an innate immune gene, and its up-regulation is strongly associated with the progression of different types of cancers. Correspondingly, it has been used as a prognostic marker for conditions like oropharyngeal cancer (48), chronic myeloid leukemia (49), and HCC (50), among others. Furthermore, in HCC, PSMD1 is found to be significantly correlated with changes in the TIME as well as immune cells (50). In addition, RAET1E, belonging to the RAET1 gene family, is classified as a major histocompatibility complex class I–related molecule (51). Earlier studies have shown that elevated levels of RAET1E expression may be linked to poor prognosis in both cervical cancer (52) and ovarian cancer (53). Conversely, TXLNA, also referred to as IL-14, is identified as a high-molecular-weight B cell growth factor, and its ectopic expression has often been linked to dismal prognostic outcomes in glioma (54). Earlier research indicates an association between TXLNA expression and the proliferative activity and low differentiation of HCC cells (55). This suggests a poor prognosis, thus leading to its use as a marker for assessing the malignancy of HCC (55). To summarize, these six IRGs that constitute our model are all related to the formation and progression of tumors to a certain degree. Thus, additional studies are necessary to explore these connections in more detail.

Throughout the progression of cancer, tumor cells undergo a gradual loss of their original differentiation phenotype and acquire certain stem-like characteristics. This transformation enables tumor cells to have stronger abilities for proliferation and migration, thus facilitating the progression of cancer (24, 56, 57). In this context, Malta et al. (24) discovered a correlation between the RNAss and the prognosis of TCGA-LIHC. Accordingly, a higher stemness score indicated a worse prognosis in terms of OS and PFS in patients with TCGA-LIHC. Our study yielded similar findings, indicating that as the patient prognosis worsened, both the SVMRS and the RNAss increased. Furthermore, they also highlighted a notable association between the RNAss and TIME (24). Therefore, we conducted an examination of the disparity in the ratio of immune cell infiltration between the HRG and LRG. Our findings indicate notable distinctions between the groups in five distinct immune cell types, namely CD4 memory T cells, monocytes, macrophages M0, mast cells, and neutrophils. Among them, CD4 memory T cells have been reported to have the ability to recognize and attack tumor cells, thereby aiding in the regulation of tumor growth and metastatic potential (58). The findings of our study show that the HRG had a greater percentage of activated T cell CD4 memory. This suggests that the HRG may be more susceptible to immunotherapy, which was confirmed during subsequent analysis. Monocytes exhibit dual roles in tumor immunity. On the one hand, monocytes have the ability to influence the TIME through different mechanisms, induce immune tolerance and angiogenesis, and increase the proliferation of tumor cells; on the other hand, monocytes can also produce antitumor effectors and activate antigen-presenting cells (59, 60). Moreover, monocytes also have the ability to differentiate into macrophages; these M0 macrophages, in their initial state, are also referred to as naive macrophages (61). Exosomes released by lung tumor cells have been documented to expedite the macrophage transformation of the M0 phenotype into the M2 phenotype, thereby promoting carcinogenic activities (62). Earlier studies have shown that patients with HCC with a high level of infiltration of macrophage M0 cells tend to have a negative prognosis (63). Furthermore, genes associated with macrophage M0 cells may offer insights into potential clinical treatment approaches for patients with HCC (63). The results of our study also revealed that the HRG, characterized by a poor prognosis, exhibited elevated levels of macrophage M0 infiltration. In addition, mast cells can facilitate the onset and progression of HCC by increasing the population of immunosuppressive cells, resulting in a poor prognosis (64). In our study, we found that the HRG had a lower proportion of mast cells in a resting state. Additionally, although not statistically significant, a higher proportion of mast cells in the HRG were in an activated state, indicating that patients in the HRG may have a poorer outcome. Additionally, the involvement of tumor-infiltrating neutrophils has been identified as a key factor in the malignant phenotypes of HCC. On the one hand, tumor-infiltrating neutrophils express a protein called PD1 ligand PDL1, which hinders the function of CD4+ and CD8+T cells by binding to PD1, and this interaction promotes the evasion of the immune system by the tumor (65–67). On the other hand, neutrophils release substances called CCL2 and CCL17, which attract immunosuppressive macrophages and Treg cells (65–67). Furthermore, the presence of both peritumoral and intratumoral neutrophils in patients with HCC has been linked to a negative prognosis. This observation implies that neutrophils may offer promising avenues for targeted therapeutic strategies (67). In our study, the HRG, which had a worse prognosis, demonstrated a greater proportion of neutrophils, aligning with the previous findings. Thus, the findings of our study are corroborated by numerous prior studies, indicating the rationality of the HRG and LRG employed in our study.

At present, the use of immunotherapy for HCC is in its initial stage, and there is a lack of definitive biomarkers to predict its effectiveness. However, the implementation of immunotherapy has shown promising advancements in the treatment outlook for advanced HCC (68–70). Hence, identifying biomarkers capable of precisely predicting immunotherapy effectiveness is expected to emerge as a prominent avenue in the treatment of HCC. Indicators, such as immune cell infiltration, PD-1/PD-L1, and tumor mutational burden/microsatellite instability in the TIME, are considered to hold considerable potential in predicting therapeutic efficacy (68–70). Our study revealed a notable increase in the PD-1/PD-L1 expression in the HRG and highlighted a positive correlation with the SVMRS. The analysis of immune cell infiltration results also indicates that the HRG may exhibit greater responsiveness to immunotherapy. Thus, we confirmed this conjecture through the analysis of patients in the three cohorts undergoing immunotherapy. Consequently, the model employed in this study was found to exhibit promising potential in predicting immunotherapy effectiveness. Further functional enrichment analysis revealed that the HRG exhibited activation of pathways associated with tumorigenesis and immune processes. This activation may contribute to the improved efficacy of immunotherapy, suggesting an internal mechanism. Among them, TLR is a pattern recognition receptor found in many different cells, which plays a crucial role in the innate immune response. Correspondingly, TLRs on tumor cells can enhance the stemness, proliferation, and metastasis of tumor cells, and resist cytotoxic lymphocyte attack (71). In HCC, the signal transduction pathway of TLRs is frequently associated with the progression (72). TLR3 and TLR4, among these receptors, hold potential as candidate prognostic indicators for treating HCC (72). Moreover, the TLR4 signaling pathway activation has been noted to foster the growth, mobility, and invasive capabilities of HCC cells, hinder programmed cell death, and accelerate resistance to tumor drugs (73). This suggests that targeting the TLR4 pathway could be a promising approach for immunotherapy in HCC. Moreover, TLR serves as a crucial link connecting the innate and acquired immune systems, playing a significant role in the body’s immune response (74). In the context of immunotherapy, the TLR signaling pathway participates in the regulation of PD-1/PD-L1 and PD-L2 expression (75). Furthermore, TLR9 agonists have also undergone extensive research for their potential use in tumor treatment, either as standalone therapies or in combination with other agents (76). In this context, the potential clinical application prospects of targeting TLR alone or in combination with other drugs have been demonstrated (77, 78). In our study, both the SVMRS and the 6 key genes of the model were significantly correlated with the majority of the key genes in the TLR signaling pathway. Additionally, pathway analysis revealed that the pathway was activated in the HRG. Thus, the activation of the TLR signaling pathway may contribute to a negative prognosis and enhance immunotherapy effectiveness in individuals at high risk. Consequently, targeting this pathway may serve as a promising therapeutic approach for this specific patient population.

Our study presents a novel approach for predicting OS and immunotherapy effectiveness for HCC using six IRG. However, there are still certain constraints that need to be acknowledged. First, the study relies on information obtained from a publicly available dataset. While we did utilize our own cohort to validate the findings, additional experimental evidence is required to definitively confirm the proposed hypothesis. However, our study revealed, via functional enrichment analysis, that the six IRGs play a role in regulating various pathways associated with tumor formation and progression. This finding is likely to provide valuable insights for future research and facilitate further investigation into the underlying molecular mechanisms. Secondly, the presence of diverse detection platforms and training methods in the datasets leads to variations in sequencing backgrounds and normalization techniques. Consequently, it becomes challenging to determine a universally applicable cut-off value for SVMRS across all datasets. Hence, it is necessary to initially acquire the threshold value of SVMRS through small sample detection prior to its application, and subsequently refine the threshold value through extensive clinical prospective studies. In order to determine immunotherapy effectiveness, it is necessary to conduct extensive prospective clinical trials to validate the use of high SVMRS as a predictive factor. Thirdly, it should be noted that the cohort of 54 patients under study did not undergo immunotherapy, thereby rendering the prediction analysis of immunotherapy response unfeasible. Meanwhile, the three cohorts used to validate immunotherapy effectiveness were all composed of patients with cancers other than HCC. Thus, further validation is required to determine if the predictive value of our model for immunotherapy efficacy in HCC cohorts is consistent.

## Conclusions

To conclude, the current study successfully resulted in the development of a prediction model for HCC using bioinformatics analysis and machine learning. This model, based on a 6-IRG signature, has the potential to accurately predict immunotherapy response. The risk score and risk groups of our model exhibited substantial variations in tumor stemness, tumor immune cell infiltration levels, ICG expression, and immunotherapy effectiveness. The key genes in our model likely participate in the regulation of various pathways associated with tumorigenesis and immune processes. Thus, our study introduces a novel approach for predicting the prognosis of HCC and evaluating immunotherapy effectiveness, providing promising prospects for clinical application.

## Data availability statement

The original contributions presented in the study are included in the article/**Supplementary Material**. Further inquiries can be directed to the corresponding authors.

## Ethics statement

The studies involving humans were approved by Clinical Research Ethics Committee of the Second Xiangya Hospital, Central South University (Approval No.: LYF2022070). The studies were conducted in accordance with the local legislation and institutional requirements. The participants provided their written informed consent to participate in this study.

## Author contributions

ZL: Conceptualization, Data curation, Formal analysis, Methodology, Software, Validation, Visualization, Writing – original draft. LY: Conceptualization, Data curation, Formal analysis, Validation, Visualization, Writing – original draft. CL: Data curation, Formal analysis, Software, Validation, Visualization, Writing – review & editing. ZW: Data curation, Formal analysis, Software, Validation, Writing – review & editing. WX: Data curation, Methodology, Validation, Visualization, Writing – review & editing. JL: Methodology, Software, Visualization, Writing – review & editing. CW: Conceptualization, Supervision, Writing – review & editing. XX: Conceptualization, Funding acquisition, Methodology, Supervision, Writing – review & editing.
