# Supplementary material for: Identification and validation of immune-related gene signature models for predicting prognosis and immunotherapy response in hepatocellular carcinoma
Source: Front Immunol. 2024 Jun 12;15:1371829. doi: 10.3389/fimmu.2024.1371829 (PMC11199539; doi:10.3389/fimmu.2024.1371829)
Supplement: Supplementary file 1 [file DataSheet_1.zip › Supplementary files/figureS5.pdf]

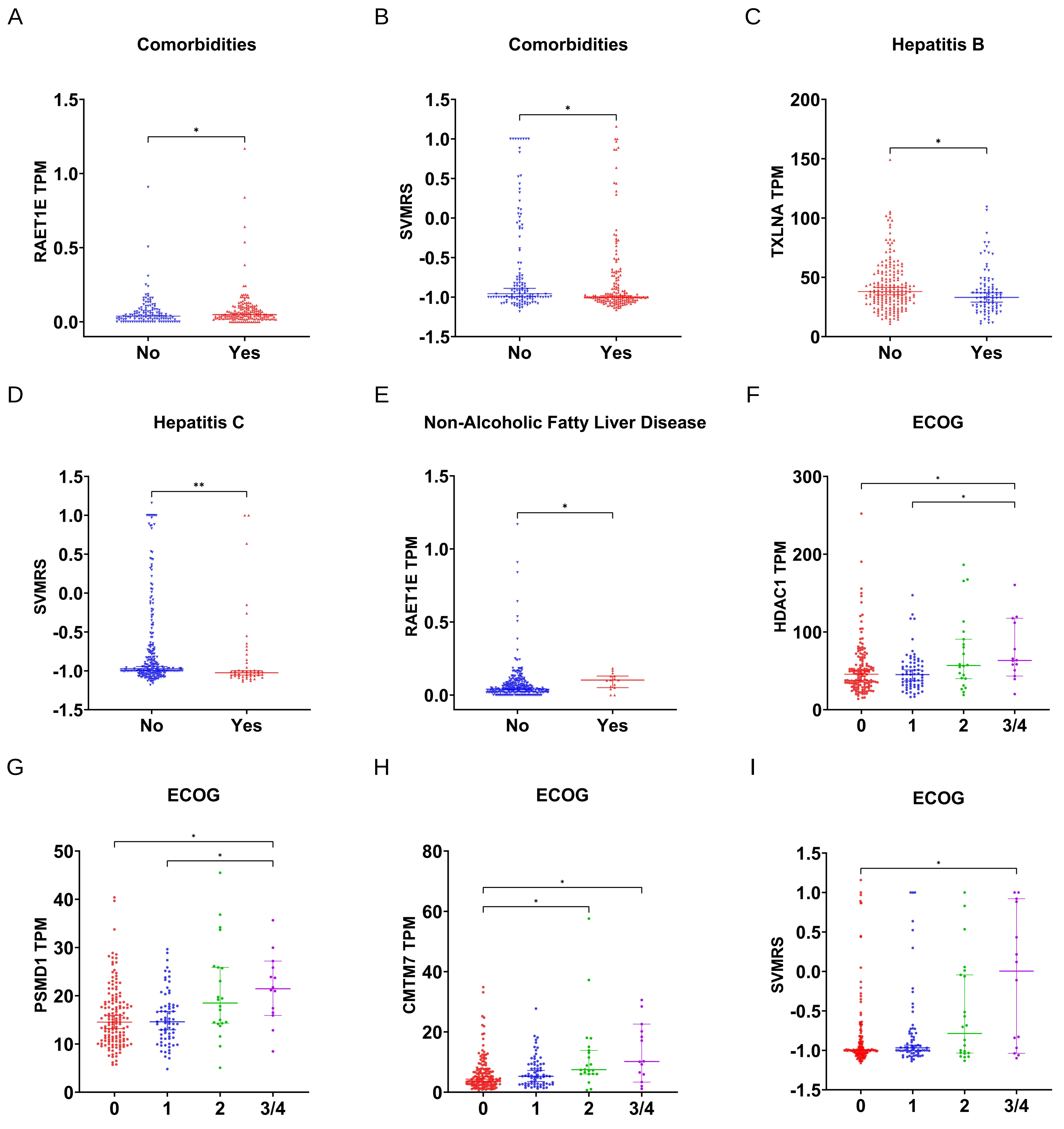

A
Comorbidities
RAET1E TPM
No
Yes
B
Comorbidities
SVMRS
No
Yes
C
Hepatitis B
TXLNA TPM
No
Yes
D
Hepatitis C
SVMRS
No
Yes
E
Non-Alcoholic Fatty Liver Disease
RAET1E TPM
No
Yes
F
ECOG
HDAC1 TPM
0
1
2
3/4
G
ECOG
PSMD1 TPM
0
1
2
3/4
H
ECOG
CMTM7 TPM
0
1
2
3/4
I
ECOG
SVMRS
0
1
2
3/4
