# Supplementary figures and images for: Identification and validation of immune-related gene signature models for predicting prognosis and immunotherapy response in hepatocellular carcinoma
Source: Front Immunol. 2024 Jun 12;15:1371829. doi: 10.3389/fimmu.2024.1371829 (PMC11199539; doi:10.3389/fimmu.2024.1371829)

PFS

OS

2033

4453

4985

81

41

253

1418

IRG

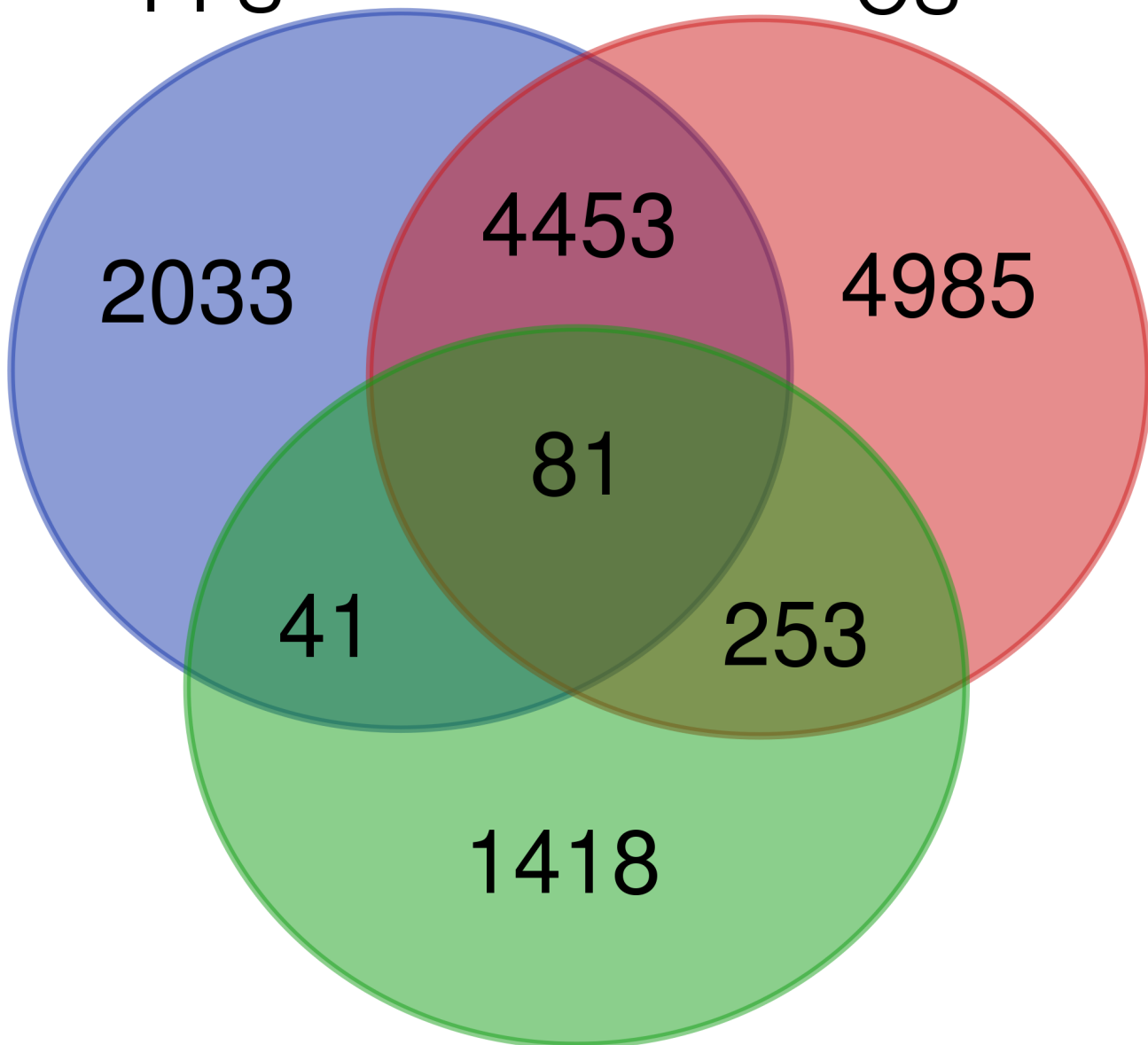

Supplement: Supplementary file 1 [file DataSheet_1.zip › Supplementary files/figureS1.pdf]

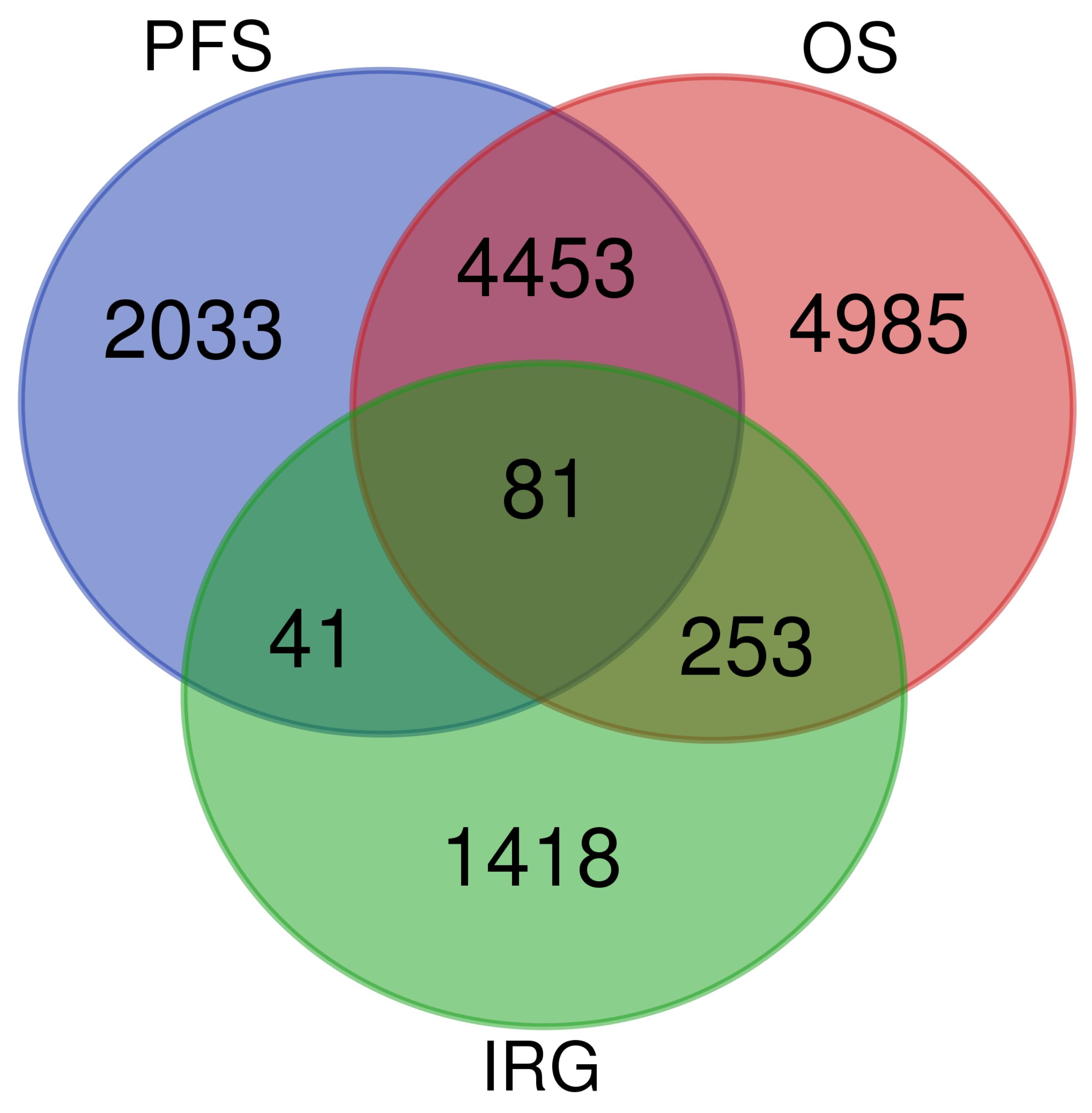

Supplement: Supplementary file 1 [file DataSheet_1.zip › Supplementary files/figureS1_00.jpg]

A

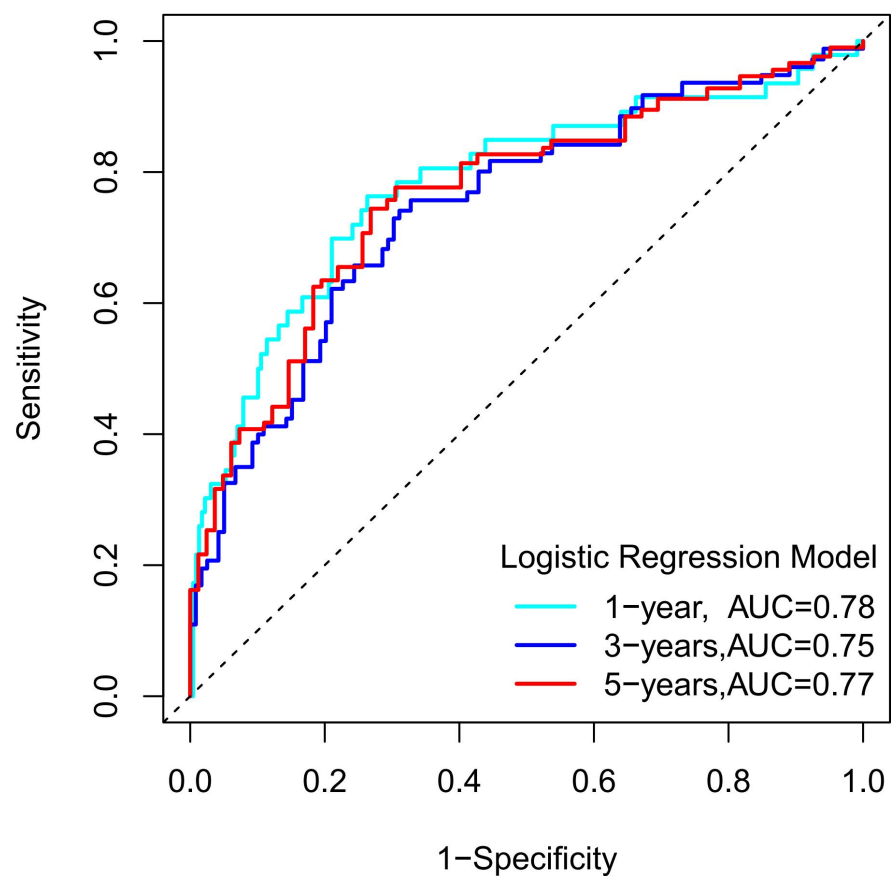

B

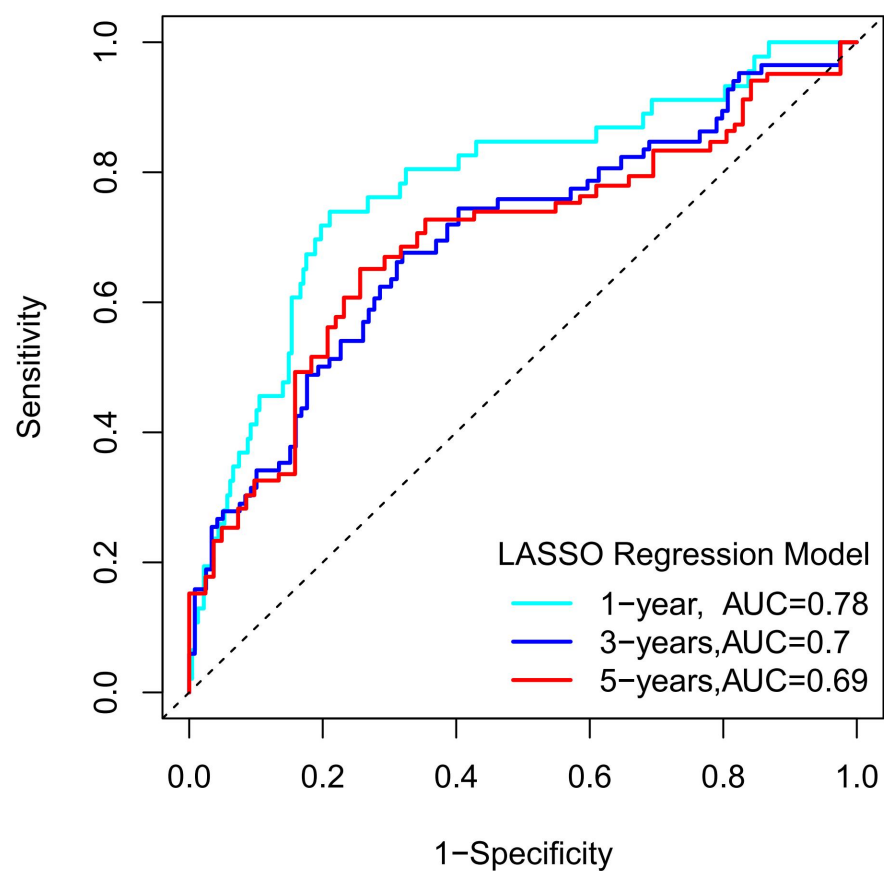

C

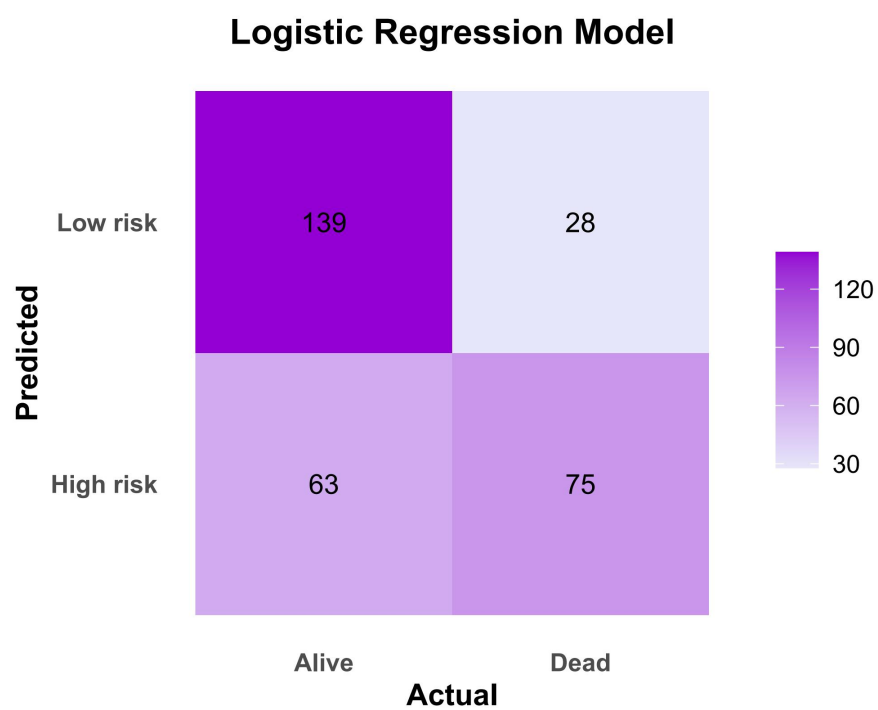

D

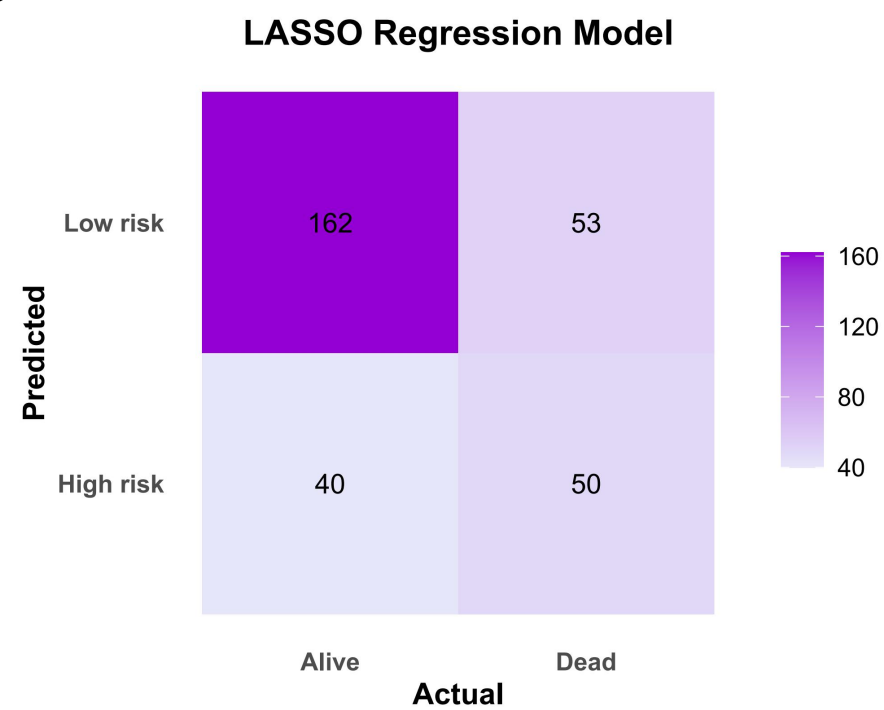

E

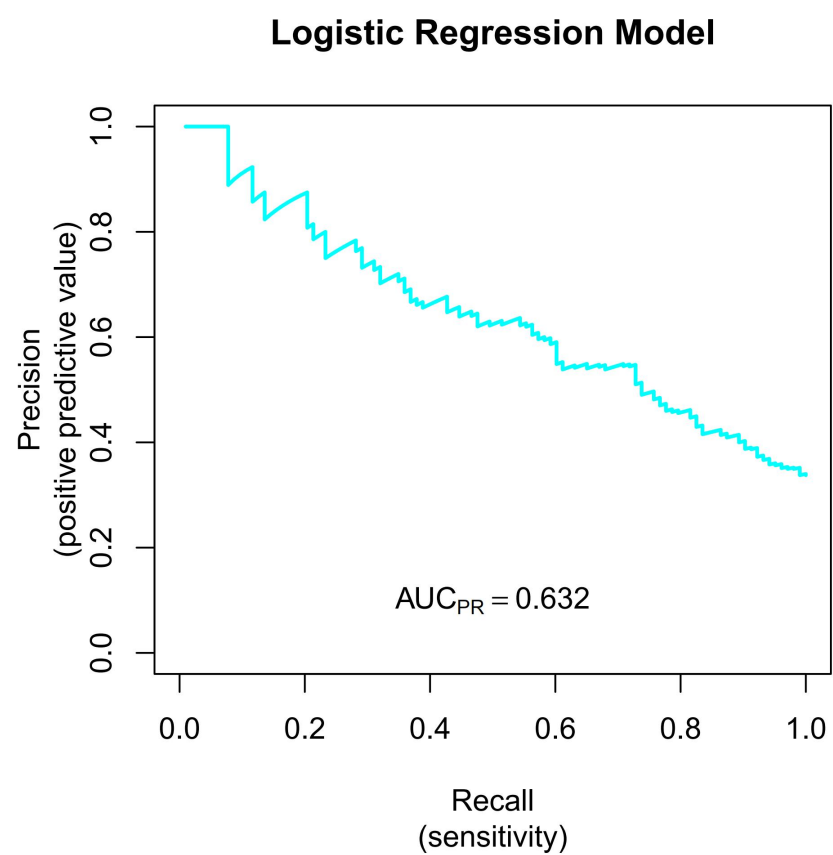

F

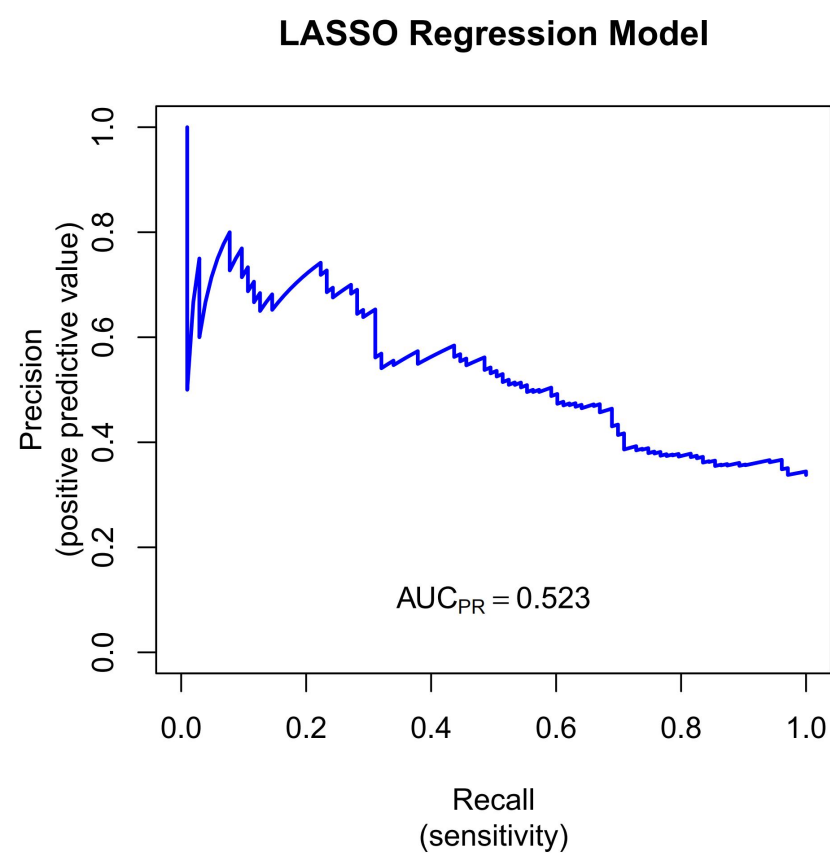

Supplement: Supplementary file 1 [file DataSheet_1.zip › Supplementary files/figureS2.pdf]

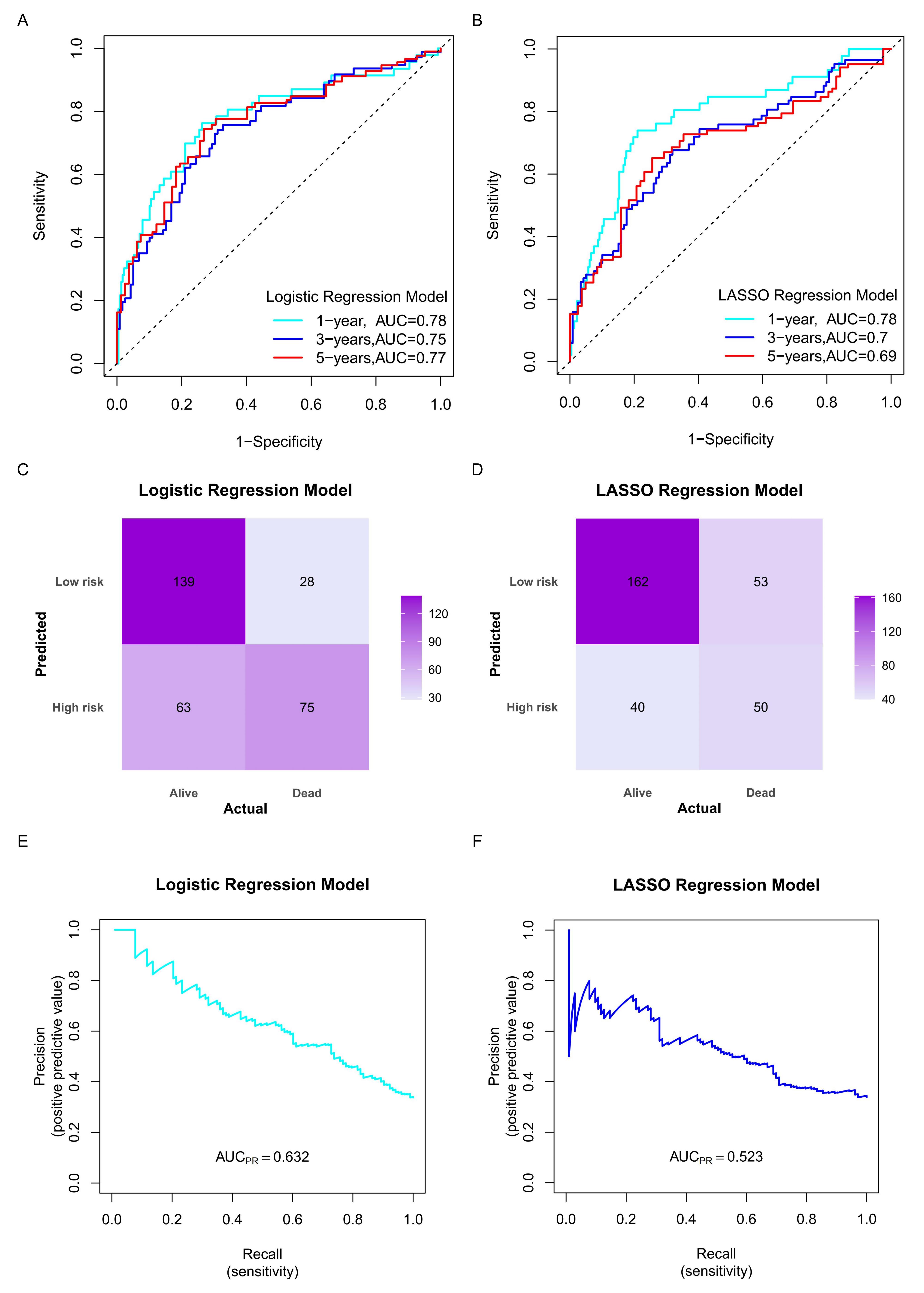

Supplement: Supplementary file 1 [file DataSheet_1.zip › Supplementary files/figureS2_00.jpg]

A

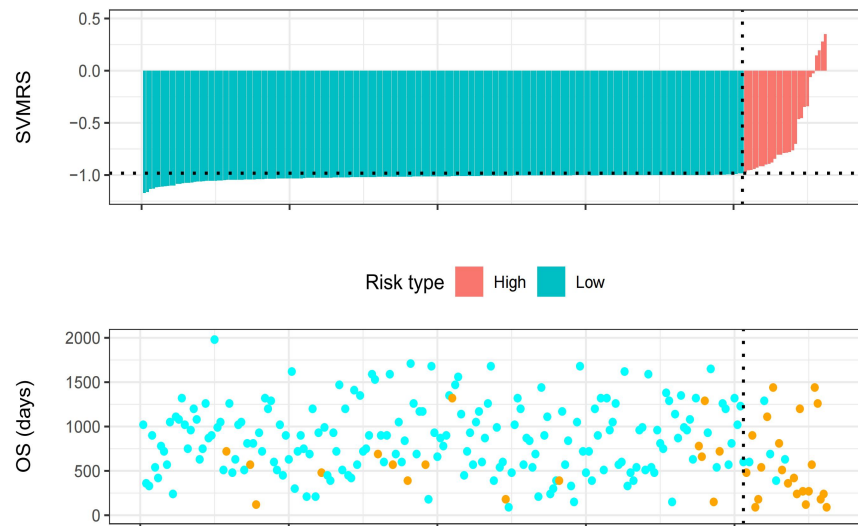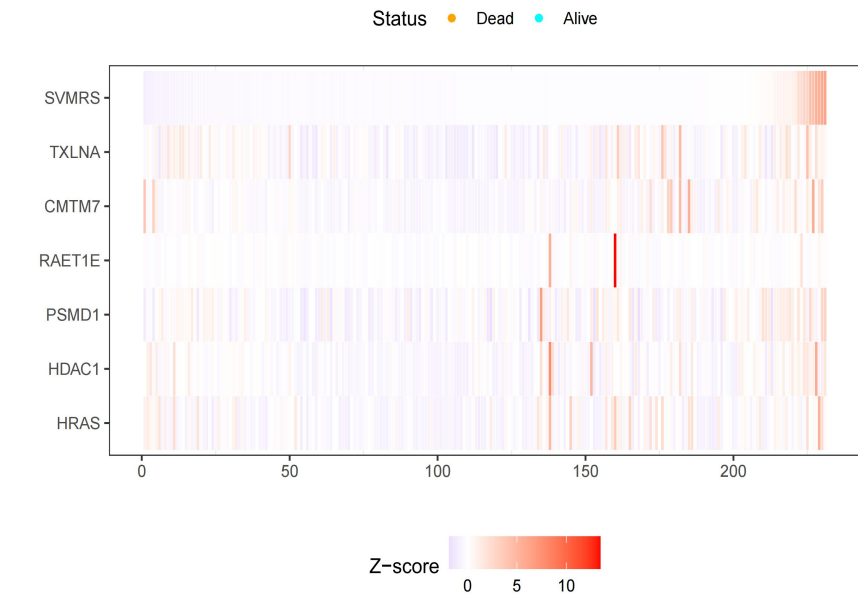

B

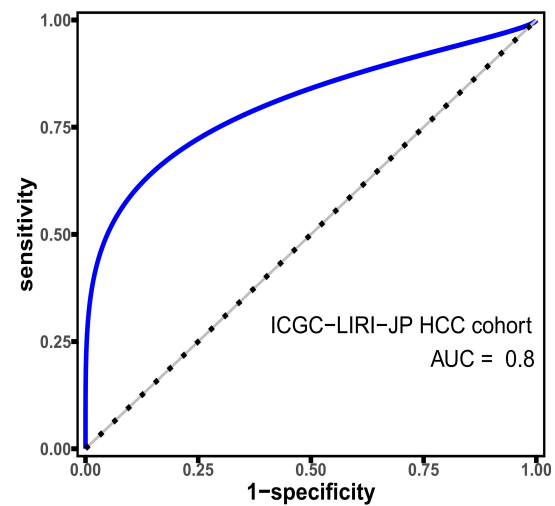

C

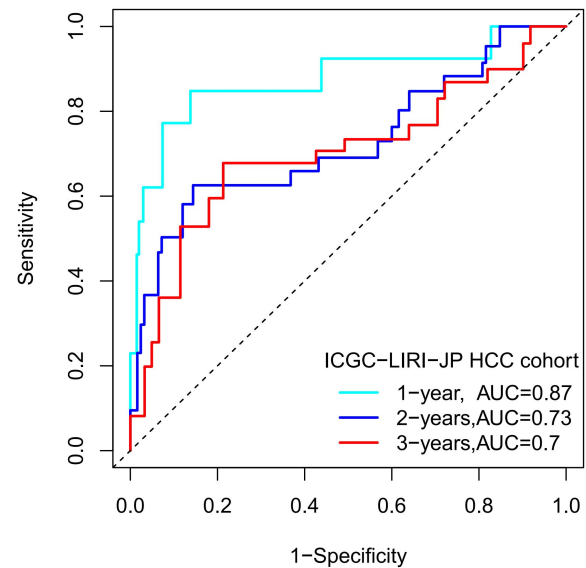

D

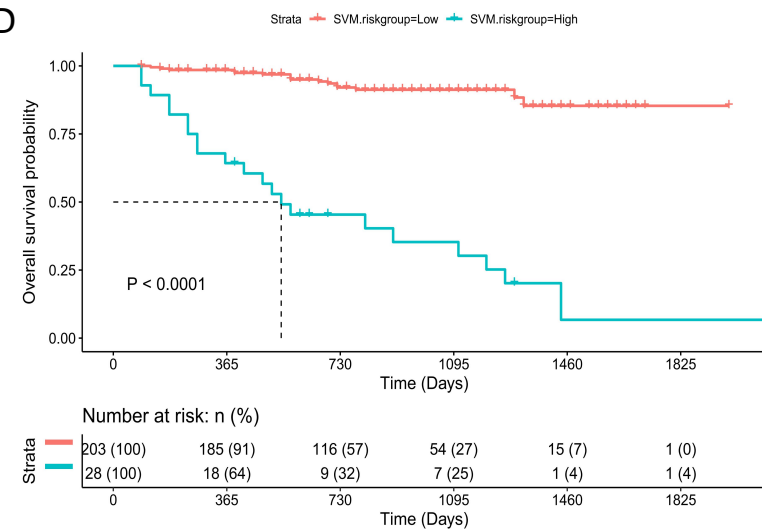

E

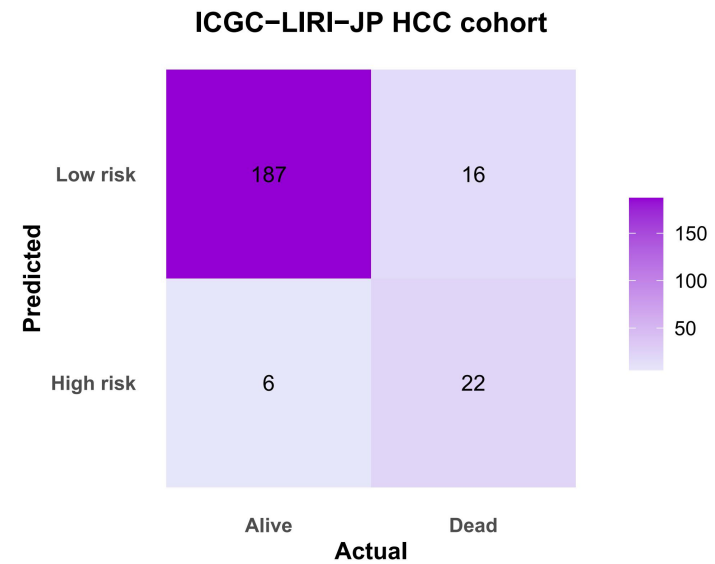

Supplement: Supplementary file 1 [file DataSheet_1.zip › Supplementary files/figureS3.pdf]

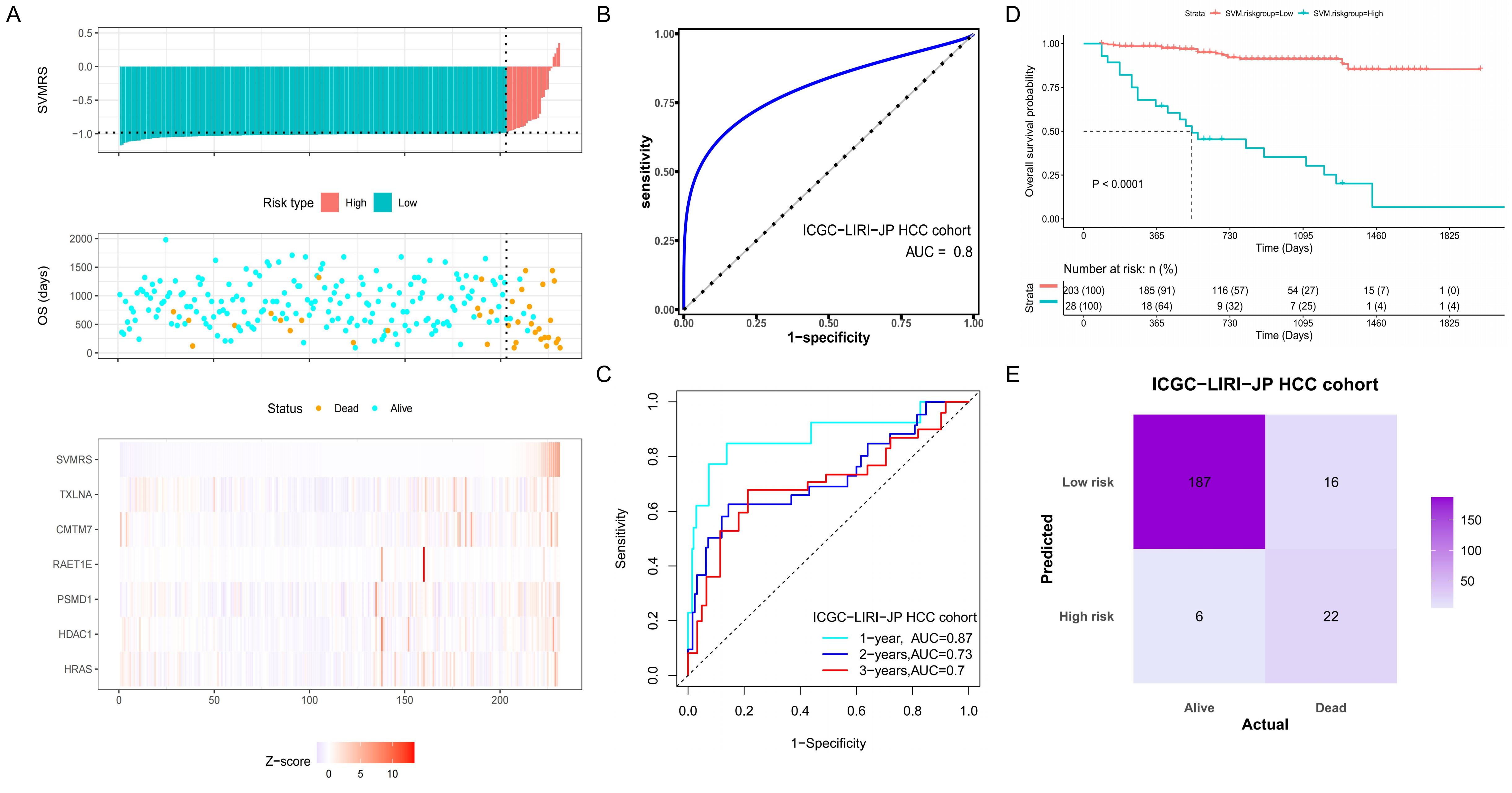

Supplement: Supplementary file 1 [file DataSheet_1.zip › Supplementary files/figureS3_00.jpg]

A

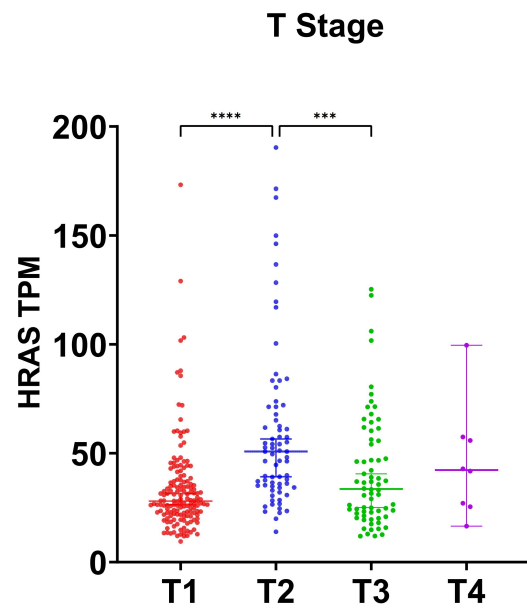

B

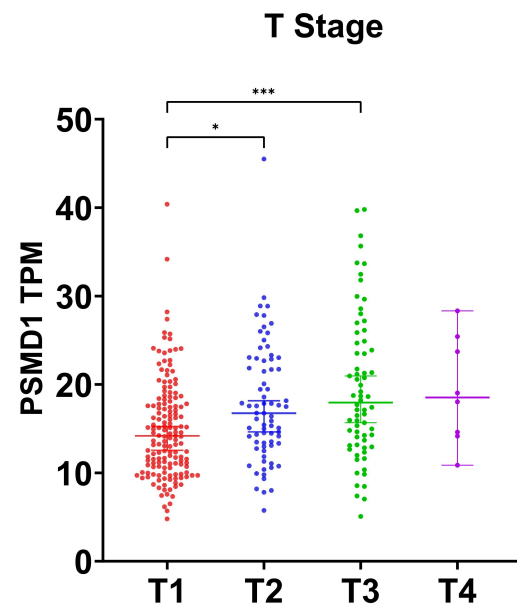

C

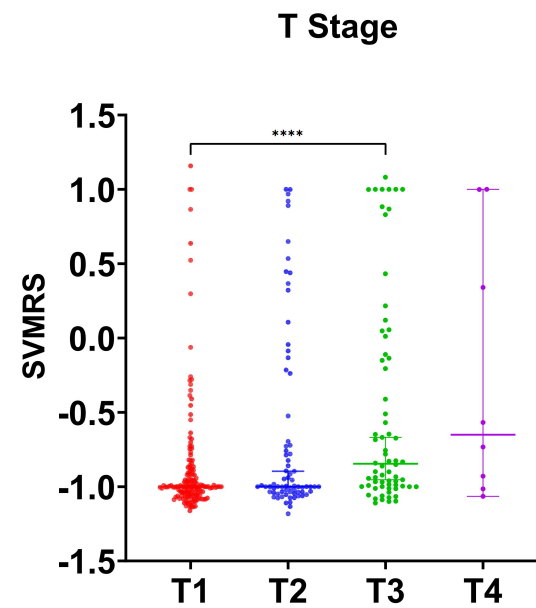

D

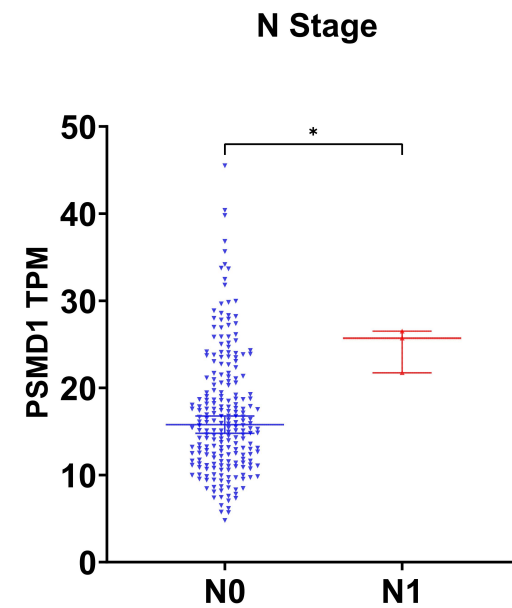

E

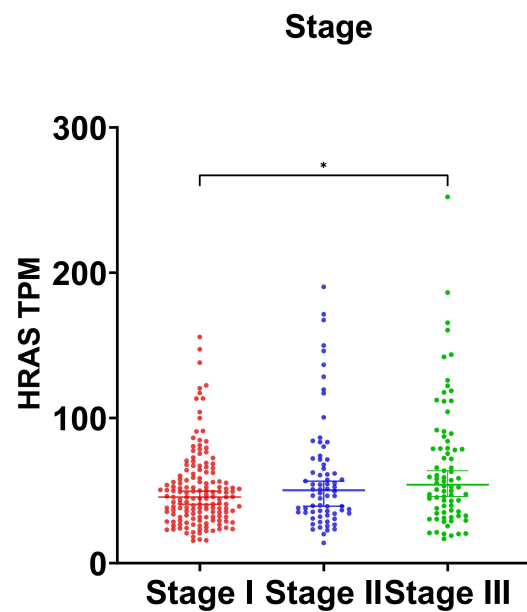

F

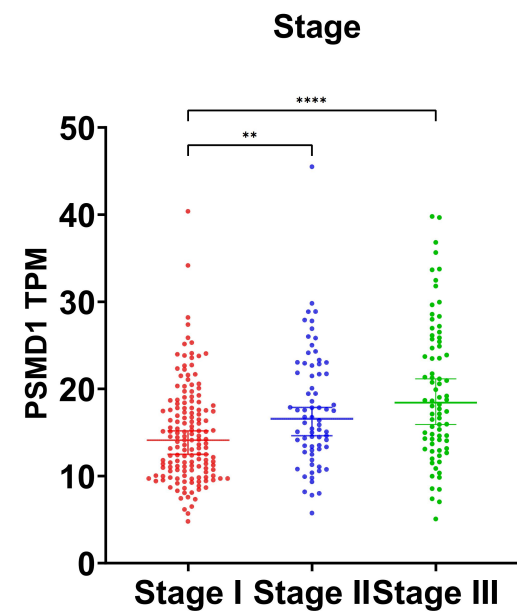

G

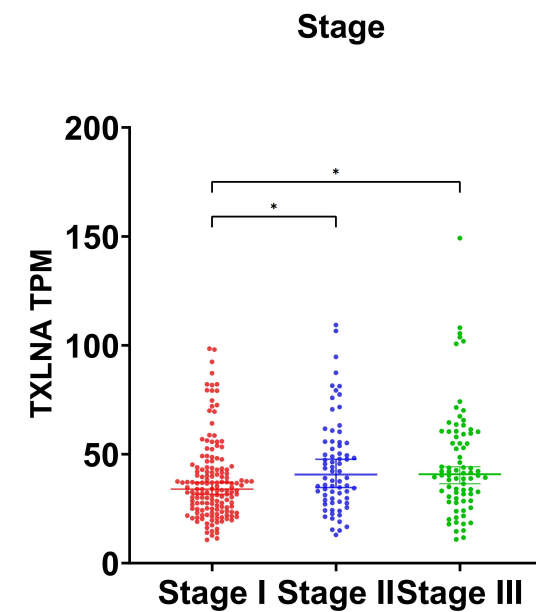

H

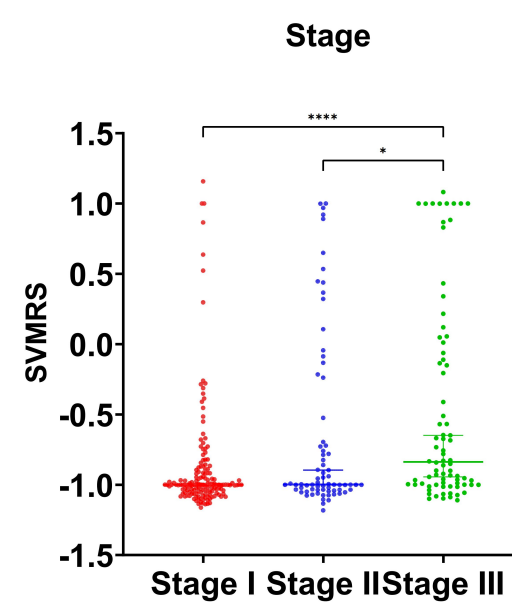

Supplement: Supplementary file 1 [file DataSheet_1.zip › Supplementary files/figureS4.pdf]

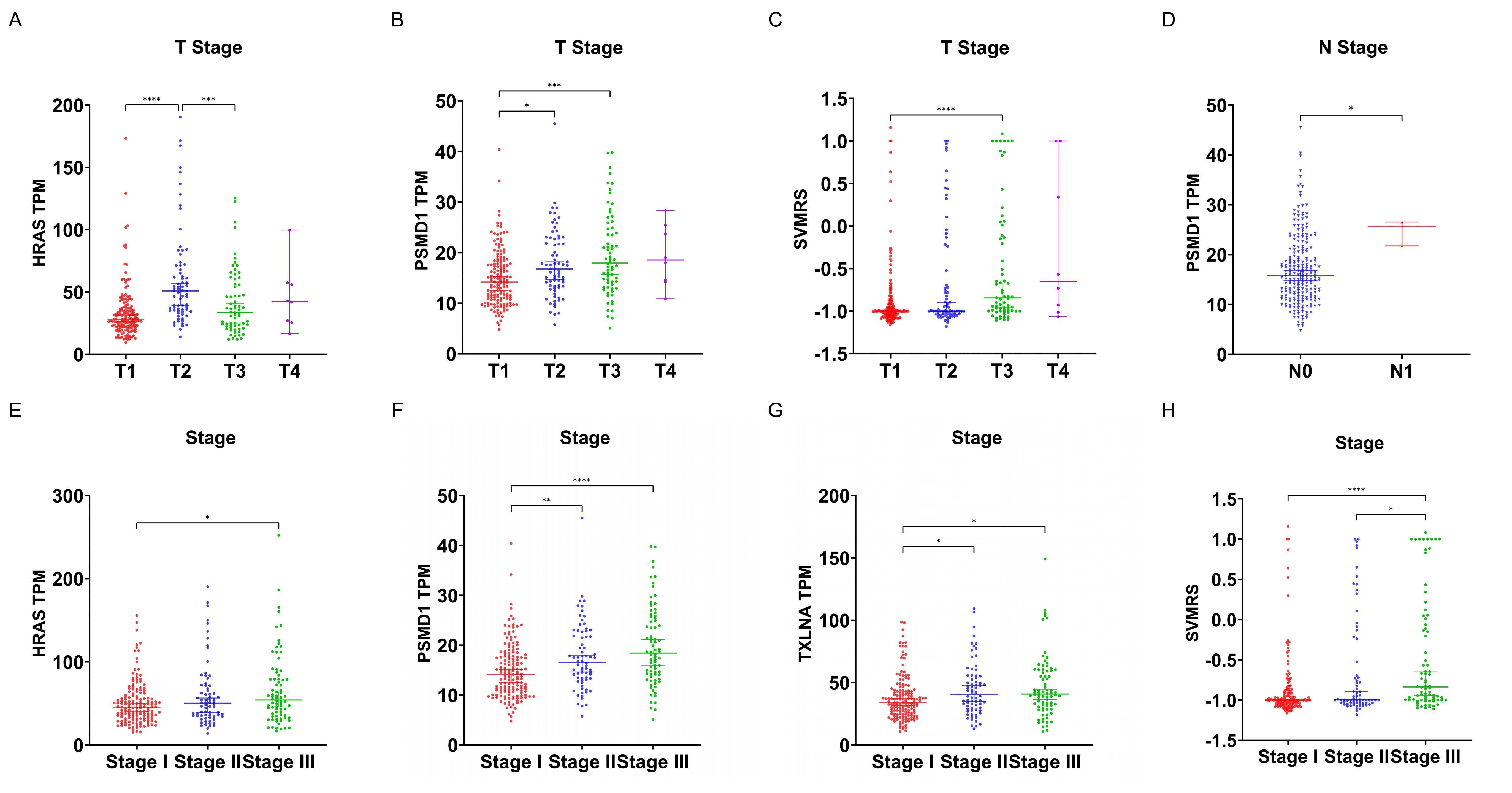

Supplement: Supplementary file 1 [file DataSheet_1.zip › Supplementary files/figureS4_00.jpg]

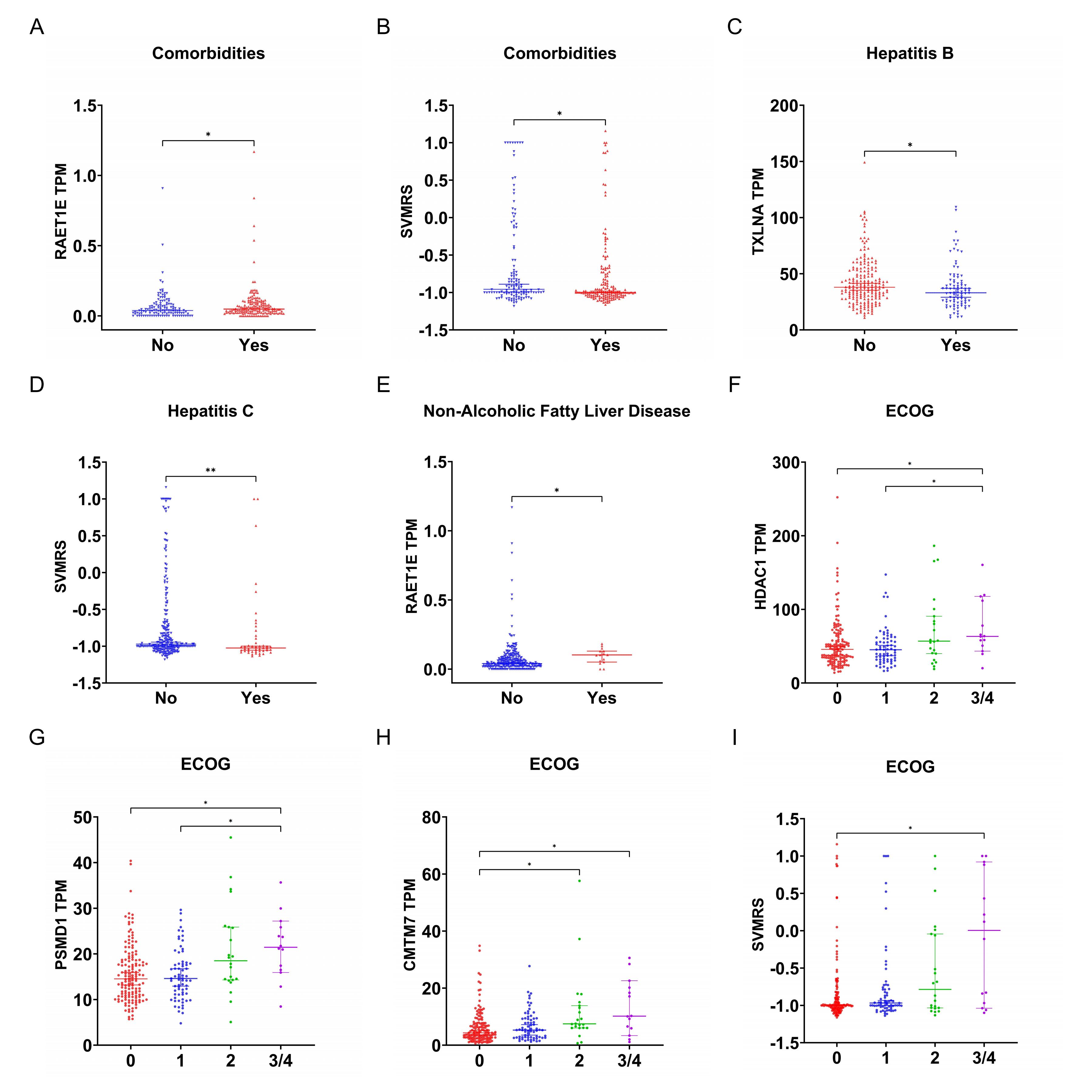

Supplement: Supplementary file 1 [file DataSheet_1.zip › Supplementary files/figureS5_00.jpg]
