# Supplementary material for: Identification and validation of immune-related gene signature models for predicting prognosis and immunotherapy response in hepatocellular carcinoma
Source: Front Immunol. 2024 Jun 12;15:1371829. doi: 10.3389/fimmu.2024.1371829 (PMC11199539; doi:10.3389/fimmu.2024.1371829)
Supplement: Supplementary file 1 [file DataSheet_1.zip › Supplementary files/TableS2.doc]

**Table S2:** Correlation analysis between SVMRS and selected clinicopathological features of 305 patients with hepatocellular carcinoma in the TCGA Database

| **Variables** | **Low risk (N = 188)** | **High risk (N = 117)** | **P value** |
| --- | --- | --- | --- |
| **T stage** |  |  |  |
| Tx/Unknown | 1 | 0 |  |
| T1 | 110 | 45 | **0.0009***** |
| T2 | 44 | 29 |  |
| T3 | 30 | 38 |  |
| T4 | 3 | 5 |  |
| **N stage** |  |  |  |
| Nx/Unknown | 46 | 27 |  |
| N0 | 141 | 87 | 0.6811 |
| N1 | 1 | 2 |  |
| **Stage** |  |  |  |
| Stage I | 111 | 44 | **0.0002***** |
| Stage II | 43 | 29 |  |
| Stage III | 34 | 44 |  |
| **Comorbidities** |  |  |  |
| No | 68 | 58 | **0.0176*** |
| Yes | 109 | 52 |  |
| Unknown | 11 | 7 |  |
| **Hepatitis B** |  |  |  |
| No | 117 | 80 | 0.2395 |
| Yes | 60 | 30 |  |
| Unknown | 11 | 7 |  |
| **Hepatitis C** |  |  |  |
| No | 143 | 98 | 0.0624 |
| Yes | 34 | 12 |  |
| Unknown | 11 | 7 |  |
| **Non-alcoholic fatty liver disease** |  |  |  |
| No | 167 | 105 | 0.6828 |
| Yes | 10 | 5 |  |
| Unknown | 11 | 7 |  |
| **ECOG performance status** |  |  |  |
| 0 | 103 | 43 | **0.0034**** |
| 1 | 44 | 25 |  |
| 2 | 10 | 12 |  |
| 3/4 | 4 | 10 |  |
| Unknown | 27 | 27 |  |

Patients were divided into high-risk group (n = 117) and low-risk group (n = 188) according to SVMRS = -0.9214. ECOG, Eastern Cooperative Oncology Group; Bold values indicate P < 0.05 and * P < 0.05, ** P < 0.01, *** P < 0.001.
